# The Application and Ethical Implication of Generative AI in Mental Health: Systematic Review

**DOI:** 10.2196/70610

**Published:** 2025-06-27

**Authors:** Xi Wang, Yujia Zhou, Guangyu Zhou

**Affiliations:** 1 School of Psychological and Cognitive Sciences Beijing Key Laboratory of Behavior and Mental Health, Key Laboratory of Machine Perception (Ministry of Education) Peking University Beijing China; 2 Department of Computer Science and Technology Tsinghua University Beijing China

**Keywords:** generative AI, mental health, large language models, mental health detection and diagnosis, therapeutic chatbots

## Abstract

**Background:**

Mental health disorders affect an estimated 1 in 8 individuals globally, yet traditional interventions often face barriers, such as limited accessibility, high costs, and persistent stigma. Recent advancements in generative artificial intelligence (GenAI) have introduced AI systems capable of understanding and producing humanlike language in real time. These developments present new opportunities to enhance mental health care.

**Objective:**

We aimed to systematically examine the current applications of GenAI in mental health, focusing on 3 core domains: diagnosis and assessment, therapeutic tools, and clinician support. In addition, we identified and synthesized key ethical issues reported in the literature.

**Methods:**

We conducted a comprehensive literature search, following the PRISMA (Preferred Reporting Items for Systematic Reviews and Meta-Analyses) 2020 guidelines, in PubMed, ACM Digital Library, Scopus, Embase, PsycInfo, and Google Scholar databases to identify peer-reviewed studies published from October 1, 2019, to September 30, 2024. After screening 783 records, 79 (10.1%) studies met the inclusion criteria.

**Results:**

The number of studies on GenAI applications in mental health has grown substantially since 2023. Studies on diagnosis and assessment (37/79, 47%) primarily used GenAI models to detect depression and suicidality through text data. Studies on therapeutic applications (20/79, 25%) investigated GenAI-based chatbots and adaptive systems for emotional and behavioral support, reporting promising outcomes but revealing limited real-world deployment and safety assurance. Clinician support studies (24/79, 30%) explored GenAI’s role in clinical decision-making, documentation and summarization, therapy support, training and simulation, and psychoeducation. Ethical concerns were consistently reported across the domains. On the basis of these findings, we proposed an integrative ethical framework, GenAI4MH, comprising 4 core dimensions—data privacy and security, information integrity and fairness, user safety, and ethical governance and oversight—to guide the responsible use of GenAI in mental health contexts.

**Conclusions:**

GenAI shows promise in addressing the escalating global demand for mental health services. They may augment traditional approaches by enhancing diagnostic accuracy, offering more accessible support, and reducing clinicians’ administrative burden. However, to ensure ethical and effective implementation, comprehensive safeguards—particularly around privacy, algorithmic bias, and responsible user engagement—must be established.

## Introduction

### Background

Mental health has become a global public health priority, with increasing recognition of its importance for individual well-being, societal stability, and economic productivity. According to the World Health Organization, approximately 1 in 8 people worldwide live with a mental health disorder [1]. Despite the growing demand for mental health services, traditional approaches such as in-person therapy and medication, which rely heavily on trained professionals and extensive infrastructure, are struggling to meet the rising need [2]. Consequently, an alarming 76% to 85% of individuals with mental health disorders do not receive effective treatment, often due to barriers such as limited access to mental health professionals, social stigma, and inadequate health care systems [3]. Against this backdrop, advances in generative artificial intelligence (GenAI) offer new and promising avenues to enhance mental health services.

GenAI, such as ChatGPT [4], is built on large-scale language modeling and trained on extensive textual corpora. Their capacity to produce contextually relevant and, in many cases, emotionally appropriate language [5,6] enables more natural and adaptive interactions. Compared to earlier dialogue systems, GenAI exhibits greater flexibility in producing open-ended, humanlike dialogue [7]. This generative capability makes them a promising tool for web-based therapeutic interventions that allow for real-time, adaptive engagement in mental health care.

Currently, GenAI is being integrated into mental health through a range of innovative applications. For instance, GPT-driven chatbots such as Well-Mind ChatGPT [8] and MindShift [9] provide personalized mental health support by engaging users in conversational therapy. Similarly, virtual companions such as Replika [10] are used to help users manage feelings of loneliness and anxiety through interactive dialogue [11]. In addition, GenAI has been used to analyze social media posts and clinical data to identify signs of depression [2] and suicidal ideation [12]. These diverse applications illustrate the potential of GenAI to address various mental health needs, from prevention and assessment to continuous support and intervention.

Although research has investigated various applications of GenAI in mental health, much of it has focused on specific models or isolated cases, lacking a comprehensive evaluation of its broader impacts, applications, and associated risks. Similarly, most systematic reviews to date have focused on particular domains, such as depression detection [13], chatbot interventions [14], empathic communication [5], psychiatric education [15], and AI-based art therapy [16]. While such focused reviews offer valuable insights into specific use cases, a broad outline remains crucial for understanding overarching trends, identifying research gaps, and informing the responsible development of GenAI in mental health. To date, only 2 reviews [17,18] have attempted broader overviews, covering the literature published before April 2024 and July 2023, respectively. However, since April 2024, the rapid evolution of GenAI—including the release and deployment of more advanced models, such as GPT-4o [19] and GPT-o1 [20], and their increasing integration with clinical workflows, such as Med-Gemini [21], has expanded the scope and complexity of GenAI applications in real-world mental health contexts. These developments underscore the need for a more updated and integrative synthesis.

### Objectives

**Figure 1:**
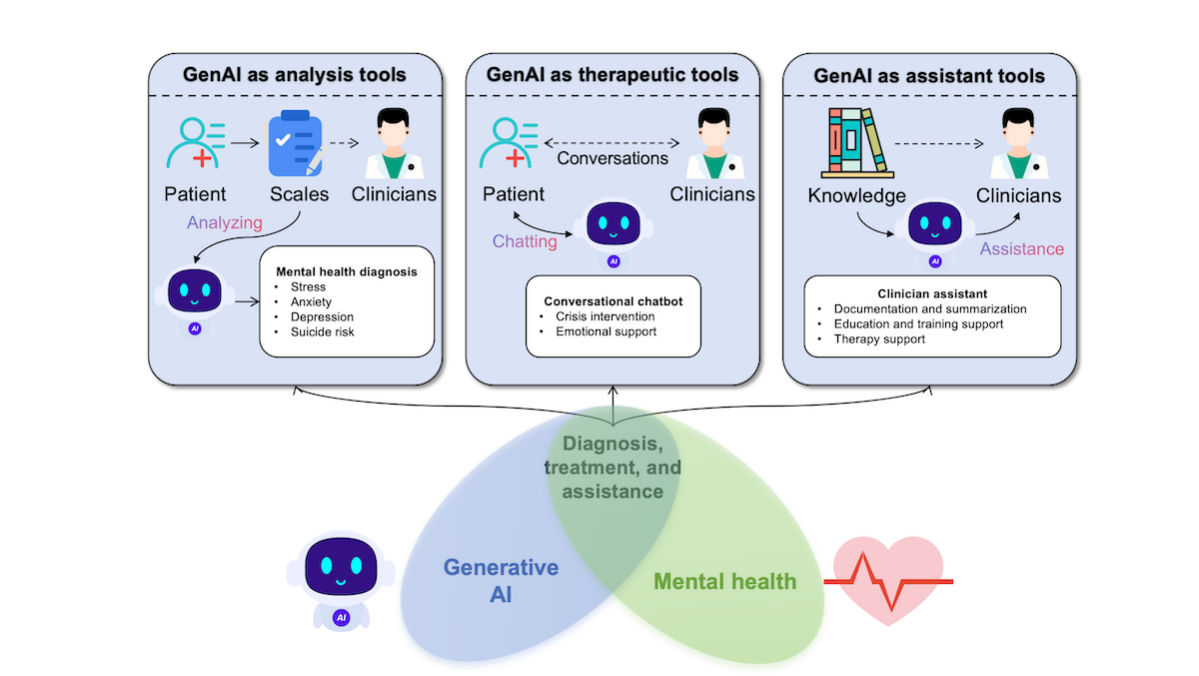
Classification of generative artificial intelligence (GenAI) applications in mental health.

To address this gap, we aimed to provide a comprehensive overview of GenAI applications in mental health, identify research gaps, and propose future directions. To systematically categorize the existing research, we divided the studies into three distinct categories based on the role of GenAI in mental health applications, as illustrated in Figure 1: (1) GenAI for mental health diagnosis and assessment, encompassing research that leverages GenAI to detect, classify, or evaluate mental health conditions; (2) GenAI as therapeutic tools, covering studies where GenAI-based chatbots or conversational agents are used to deliver mental health support, therapy, or interventions directly to users; and (3) GenAI for supporting clinicians and mental health professionals, including research aimed at using GenAI to assist clinicians in their practice.

**Figure 2:**
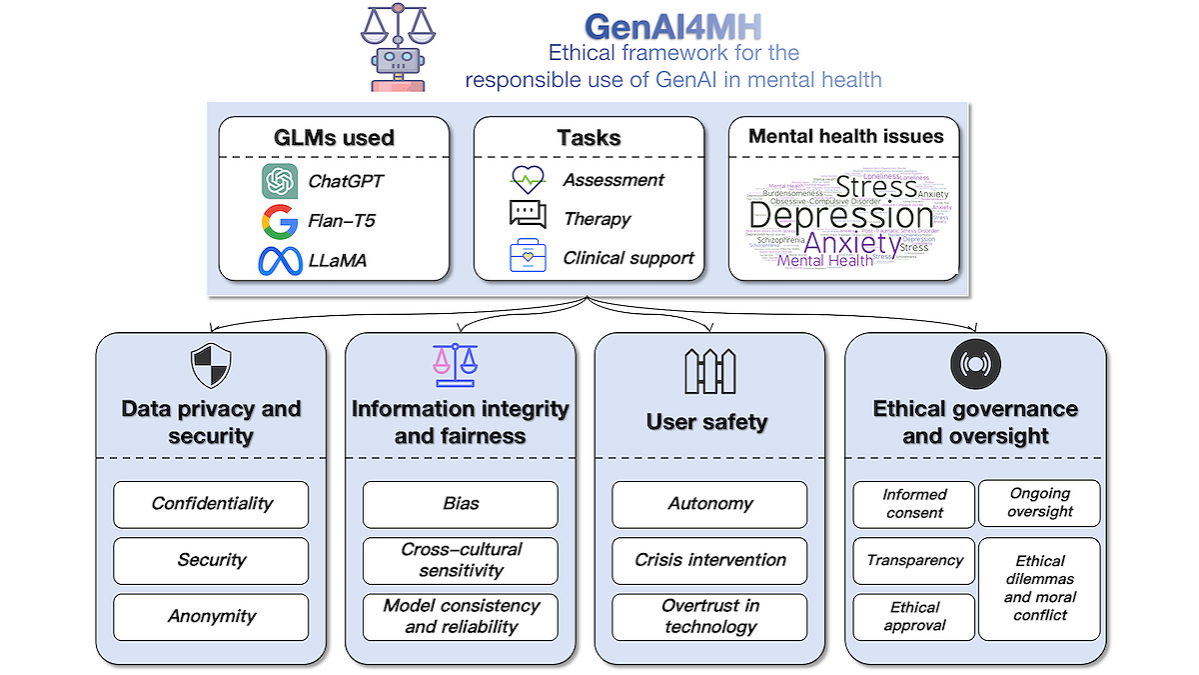
Overview of the GenAI4MH ethical framework for the responsible use of generative artificial intelligence (GenAI) in mental health. GLM: generative language model.

Despite these promising applications, the integration of GenAI into mental health care is not without challenges. Applying GenAI in the mental health field involves processing highly sensitive personal information, such as users’ emotional states, psychological histories, and behavioral patterns. Mishandling such data not only poses privacy risks but may also lead to psychological harm, including distress, stigma, or reduced trust in mental health services [22]. Therefore, in addition to systematically categorizing existing applications of GenAI in mental health, we also examined ethical issues related to their use in this domain. On the basis of our analysis, we proposed an ethical framework, GenAI4MH, to guide the responsible use of GenAI in mental health contexts (Figure 2).

## Methods

### Search Strategy

We conducted this systematic review following the PRISMA (Preferred Reporting Items for Systematic Reviews and Meta-Analyses) 2020 guidelines (Multimedia Appendix 1) [23]. We conducted a comprehensive search across 6 databases: PubMed, ACM Digital Library, Scopus, Embase, PsycInfo, and Google Scholar. We conducted the search between October 1, 2024, and October 7, 2024, and targeted studies published from October 1, 2019, to September 30, 2024. The starting date was chosen to coincide with the introduction of the T5 model [24], a foundational development for many of today’s mainstream GenAI models. This date also intentionally excluded earlier models, such as Bidirectional Encoder Representations from Transformers (BERT) [25] and GPT-2 [26], as these models have already been extensively covered in the previous literature [27,28], and our aim was to highlight more recent innovations.

Search terms were constructed using a logical combination of keywords related to GenAI and mental health: (Generative AI OR Large Language Model OR ChatGPT) AND (mental health OR mental disorder OR depression OR anxiety). This search string was developed based on previous reviews and refined through iterative testing to ensure effective identification of relevant studies. When possible, the search was restricted to titles and abstracts. For Google Scholar, the first 10 pages of results were screened for relevance. A detailed search strategy is provided in Multimedia Appendix 2.

### Study Selection

The selection criteria included studies that (1) used GenAI and were published after the introduction of the T5 [24] model and (2) directly addressed the application of GenAI in mental health care settings. Only peer-reviewed original research articles were considered, with no language restrictions.

### Data Extraction

Data from the included studies were extracted using standardized frameworks. For qualitative studies, we used the Sample, Phenomenon of Interest, Design, Evaluation, and Research Type (SPIDER) framework. For quantitative studies, we applied the Population, Intervention, Comparison, Outcome, and Study (PICOS) framework. A summary of the extracted data are provided in Multimedia Appendix 3 [2,3,7-9,11,12,29-100].

### Reporting Quality Assessment

To assess the reporting transparency and the methodological rigor of the included studies, we applied the Minimum Information about Clinical Artificial Intelligence for Generative Modeling Research (MI-CLAIM-GEN) checklist (Multimedia Appendix 4) [101], a recently proposed guideline tailored for evaluating the reporting quality of research on GenAI in health care. The checklist covers essential aspects such as study design, data and resource transparency, model evaluation strategies, bias and harm assessments, and reproducibility. We followed the Joanna Briggs Institute quality appraisal format [102] to score each item in the checklist using 4 categories: yes, no, unclear, and not applicable.

## Results

### Study Selection

**Figure 3:**
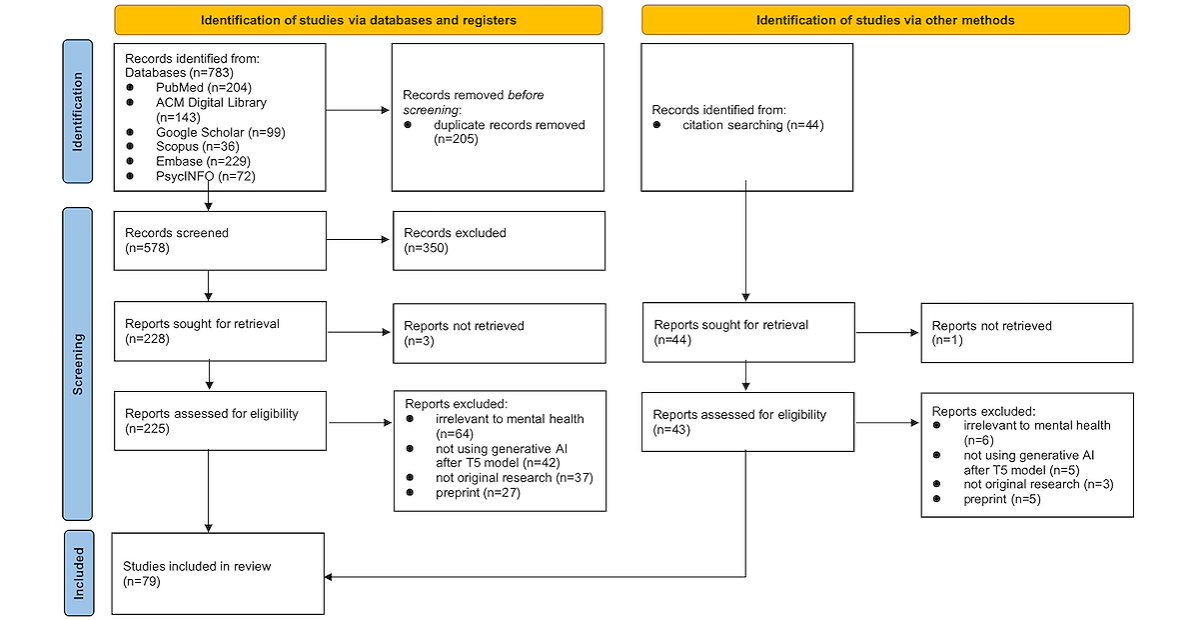
The PRISMA (Preferred Reporting Items for Systematic Reviews and Meta-Analyses) flow diagram of study selection.

As shown in Figure 3, a total of 783 records were initially retrieved from the 6 databases. After removing duplicates, 73.8% (578/783) of unique records remained for screening. Following abstract screening, 39.4% (228/578) of the records were identified for full-text retrieval and screening. After full-text screening, 24% (55/228) of the articles were selected for inclusion in the systematic review. To ensure comprehensive coverage of relevant studies, a snowballing technique was then applied, where we examined the reference lists of the included studies and related review articles. This process identified an additional 44 studies for eligibility assessment. After the same evaluation process, 54% (24/44) of these studies met the inclusion criteria, bringing the final total to 79 studies for the systematic review. Two PhD candidates (YZ and XW) independently conducted the selection, with discrepancies resolved through discussion. The interrater reliability was satisfactory (κ=0.904).

### Publication Trends Over Time

**Figure 4:**
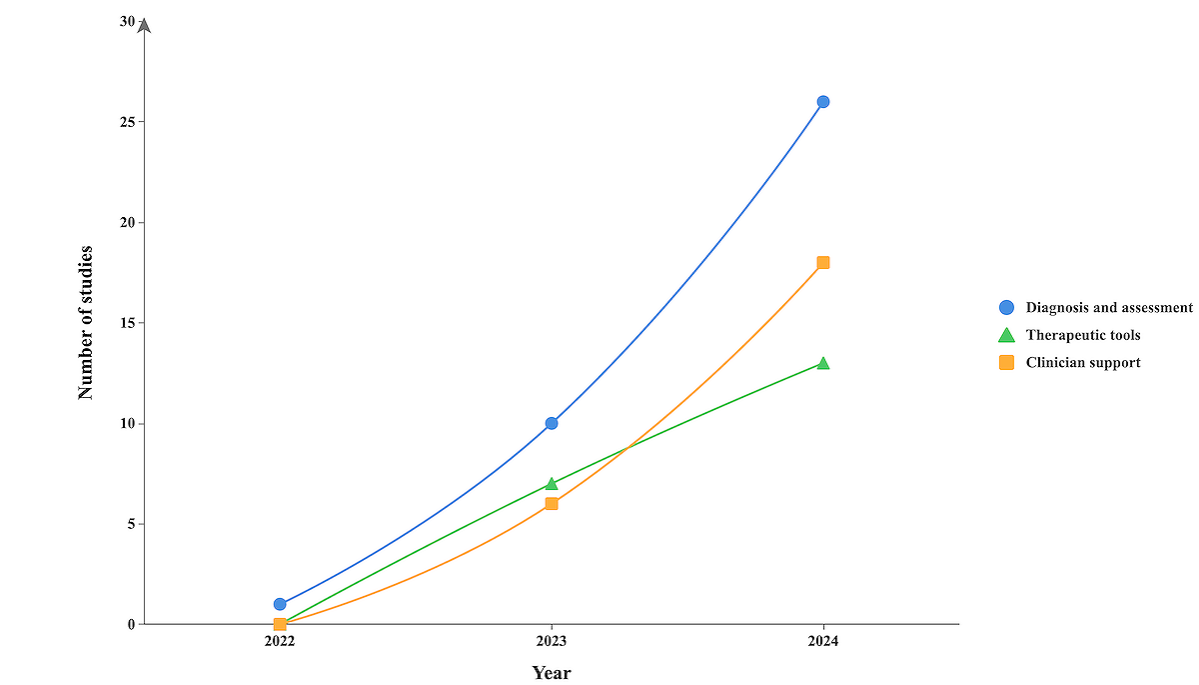
Publication trends in the application of generative artificial intelligence (GenAI) in mental health research.

An analysis of publication trends over time reveals a growing focus on the application of GenAI in mental health (Figure 4). Overall, the number of studies in all the 3 categories grew extensively over the examined period, indicating a rising interest in using GenAI for mental health. In 2022, the total number of studies was minimal across all the 3 categories, with only 1 (1%) early study, of the included 79 studies, emerging on the use of GenAI for mental health diagnosis and assessment. However, as GenAI advanced and garnered wider adoption, the number of publications in all the 3 categories began to increase steadily. A moderate increase was observed in the year 2023, with 13% (10/79) of the studies focused on diagnosis and assessment, 9% (7/79) on therapeutic interventions, and 8% (6/79) on clinician support, reflecting a growing interest in practical applications of these models in health care settings. By 2024, the number of publications had surged across all the 3 categories, with 33% (26/79) of the studies focused on diagnosis and assessment, 16% (13/79) on therapeutic interventions, and 23% (18/79) on clinician support.

### GenAI for Mental Health Diagnosis and Assessment

#### Overview

Of the 79 included studies, 37 (47%) were identified that investigated the effectiveness and applications of GenAI in mental health diagnosis and assessment. These studies primarily explored how GenAI can detect and interpret mental health conditions by analyzing textual and multimodal data. A summary of the included studies is presented in Multimedia Appendix 5 [2,3,12,29-59,61,62,100].

#### Mental Health Issues

The existing studies using GenAI for mental health diagnosis predominantly focused on suicide risk and depression, followed by emerging applications in emotion recognition, psychiatric disorders, and stress.

Suicide risk was the most frequently examined topic, addressed in 40% (15/37) of the studies. Researchers used large language models (LLMs) to identify suicide-related linguistic patterns [12], extract and synthesize textual evidence supporting identified suicide risk levels [29-33,103], and evaluate suicide risk [34-41]. GenAI models, such as GPT-4 [104], achieved high precision (up to 0.96) in predicting suicidal risk levels [30-33,103], outperforming traditional models, such as support vector machines (SVM) [41], and performing comparably to or better than pretrained language models, such as BERT [38,40]. Most studies (13/15, 87%) relied on simulated case narratives [34,36,37] or social media data [38-40]; only 13% (2/15) of the studies used real clinical narratives [35,41].

Depression was the second most common mental health issue addressed, featured in 35% (13/37) of the studies. While GenAI models showed promising accuracy (eg, 0.902 using semistructured diaries [42]), performance was often constrained to English data [43,44], with notable drop-offs in dialectal or culturally divergent contexts [44]. Multimodal approaches—integrating audio, visual, and physiological data—improved detection reliability over text-only methods [45-47]. Several studies (3/13, 23%) also explored interpretability, using GenAI to generate explanations [43] or conduct structured assessments [48].

GenAI has also been explored for emotion recognition, using smartphone and wearable data to predict affective states with moderate accuracy [47,49], and enabling novel assessment formats, such as virtual agent interactions [45] and conversational psychological scales [50]. The studies also explored other psychiatric disorders, such as obsessive-compulsive disorder (accuracy up to 96.1%) [51] and schizophrenia (*r*=0.66-0.69 with expert ratings) [52]. In total, 8% (3/37) of the studies addressed stress detection from social media texts [39,53,54].

A smaller set of studies (3/37, 8%) assessed GenAI models’ capacity for differential diagnosis, demonstrating that GenAI models could distinguish among multiple mental disorders in controlled simulations [3,55,56]. However, performance remained higher for mental health conditions with distinct symptoms (eg, psychosis and anxiety) and lower for overlapping or less prevalent disorders (eg, perinatal depression and lysergic acid diethylamide use disorder) [56], particularly for those with symptom overlap with more common mental health conditions (eg, disruptive mood dysregulation disorder and acute stress disorder) [55].

#### Model Architectures and Adaptation Strategies

##### Overview

Most included studies (29/37, 78%) used proprietary GenAI models for mental health diagnosis and assessment, with GPT-based models (GPT-3, 3.5, and 4) [4] being the most commonly used [2,3,12]. Other proprietary models included Gemini [49,51] and the pathways language model (version 2) [47]. A smaller subset of the studies (14/37, 38%) adopted open-source models, such as LLM Meta AI (LLaMA) [29,30,32,40,52,57,58], Mistral [33], Falcon [40], and Neomotron [55]. Beyond model selection, several studies (29/37, 78%) explored technical strategies to enhance diagnostic performance and interpretability. In total, 3 main approaches were identified as described in subsequent sections.

##### Hybrid Modeling

A limited number of studies (2/37, 5%) explored hybrid architectures, combining GenAI-generated embeddings with classical classifiers, such as SVM or random forest [43,53]. For example, Radwan et al [53] used GPT-3 embeddings to generate text vectors, which were input into classifiers, such as SVM, random forest, and k-nearest neighbors, for stress level classification. The combination of GPT-3 embeddings with an SVM classifier yielded the best performance, outperforming other hybrid configurations and traditional models such as BERT with the long short-term memory model.

##### Fine-Tuning and Instruction Adaptation

Some studies (4/37, 11%) used instruction-tuned models, including Flan [39,41], Alpaca [39], and Wizard [32], to enhance instruction following. Further fine-tuning with mental health–related data was also applied to improve diagnostic and assessment capabilities [39,46,59]. For instance, Xu et al [39] demonstrated that their fine-tuned models—Mental-Alpaca and Mental-FLAN-T5—achieved a 10.9% improvement in balanced accuracy over GPT-3.5 [4], despite being 25 and 15 times smaller, respectively. These models also outperformed GPT-4 [104] by 4.8%, although GPT-4 is 250 and 150 times larger, respectively.

##### Prompt Engineering and Knowledge Augmentation

Prompt-based techniques—including few-shot learning [31,39,40,46,54,58], chain-of-thought prompting [42,47,49,59,60], and example contrast [54]—have been shown to substantially enhance diagnostic performance, especially for smaller models [39]. Meanwhile, retrieval-augmented generation (RAG) approaches enriched LLMs with structured knowledge (eg, *Diagnostic and Statistical Manual of Mental Disorders, Fifth Edition* criteria), improving factual grounding in some cases [47], but occasionally introducing noise or reducing performance due to redundancy and semantic drift [55].

#### Data Source

Table 1 summarizes the datasets used for GenAI-based mental health diagnosis and assessment, categorized by data modality and mental health focus. The full dataset list, including metadata and sampling details, is provided in Multimedia Appendix 6 [12,30,35,41,42,44,49,53,56,62,103,105-133].

Social media posts, such as those on Reddit [12,29,43], Twitter [58], and Weibo [45], emerged as prominent data sources. Beyond social media, 19% (7/37) of the studies used professionally curated clinical vignettes, providing controlled scenarios that simulate clinical cases and allow for standardized assessment across GenAI models [34,51]. Only a few studies (4/37, 11%) used clinical text data sources, including clinical interviews [61], diary texts [42], and written responses of participants [50,62].

In total, 14% (5/37) of the studies used multimodal data sources—such as speech [44,45], sensor data [47], and electroencephalogram (EEG) [46]—to enhance the accuracy and comprehensiveness of mental health assessments. For example, Englhardt et al [47] developed prompting strategies for GenAI models to classify depression using passive sensing data (eg, activity, sleep, and social behavior) from mobile and wearable devices, achieving improved classification accuracy (up to 61.1%) over classical machine learning baselines. Similarly, Hu et al [46] integrated EEG, audio, and facial expressions to boost predictive performance and proposed MultiEEG-GPT, a GPT-4o-based method for mental health assessment using multimodal inputs, including EEG, facial expressions, and audio. Their results across the 3 datasets showed that combining EEG with audio or facial expressions significantly improved prediction accuracy in both zero-shot and few-shot settings.

**Table 1:** Summary of datasets used in the studies on generative artificial intelligence (GenAI) models for mental health diagnosis and assessment.

Categories			References
**By modality**			
	Text (clinical vignettes)	[56,105-108]	
	Text (social media posts)	[12,40,53,109-123,134]	
	Text (transcripts)	[35,124]	
	Text (daily self-reports)	[42,62]	
	Multimodal dataset	[44,49,125-131]	
**By mental health issues**			
	Depression	[42,44,62,109,110,113,114,117,123,126-128,130,134]	
	Suicide risk	[12,35,40-42,108,109,111,112,118,119,121,122]	
	Posttraumatic stress disorder	[110,125,127]	
	Anxiety	[115,125,128]	
	Bipolar disorder	[120,124]	
	Stress	[53,115,132]	
	Emotion regulation	[62,129,131]	
	Multiple psychiatric disorders	[56,63,106,107,133]	

### GenAI as Therapeutic Tools

Of the 79 included studies, 20 (25%) investigated the use of GenAI-based chatbots and conversational agents to facilitate interventions ranging from emotional support to more structured therapies. To assess the feasibility and potential impact of these interventions, we analyzed studies across four key dimensions: (1) therapeutic targets, (2) implementation strategies, (3) evaluation outcomes, and (4) real-world deployment features.

#### Intervention Targets and Theoretical Alignments

**Figure 5:**
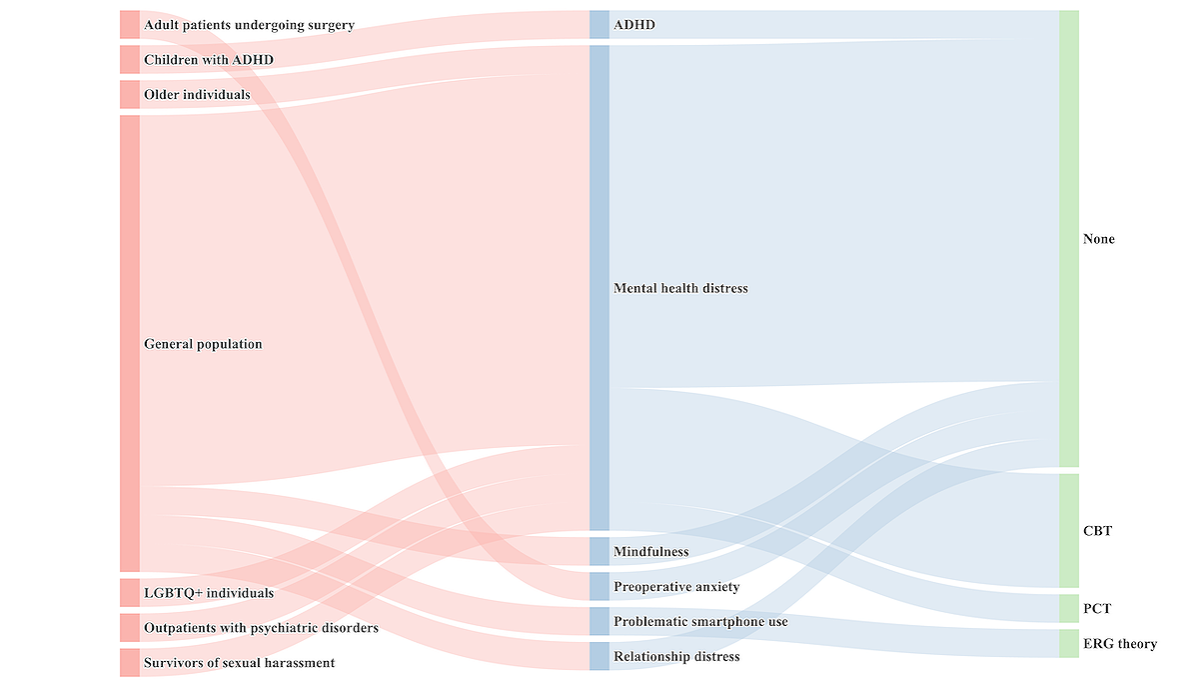
Sankey diagram mapping target group, problem, and theoretical framework in generative artificial intelligence–based mental health therapy research. ADHD: attention-deficit/hyperactivity disorder; CBT: cognitive behavioral therapy; ERG: existence, relatedness, and growth; LGBTQ+: lesbian, gay, bisexual, transgender, queer, and other minority groups; PCT: present‐centered therapy.

As presented in Figure 5, most studies (16/20, 80%) targeted the general population. A smaller subset (5/20, 25%) focused on vulnerable or underserved groups, including outpatients undergoing psychiatric treatment [64], lesbian, gay, bisexual, transgender, and queer (LGBTQ) individuals [65], sexual harassment survivors [66], children with attention-deficit/hyperactivity disorder [67], and older adults [68]. In addition to population-specific adaptations, some studies (4/20, 20%) focused on chatbots targeting specific psychological and behavioral challenges, including attention-deficit/hyperactivity disorder [67], problematic smartphone use [69], preoperative anxiety [70], and relationship issues [71].

Despite the growing prevalence of these systems, most studies do not explicitly state the theoretical frameworks guiding their development. Among the reviewed studies, only 30% (6/20) of the studies explicitly adopted a psychological theory: person-centered therapy [72]; cognitive behavioral therapy [7,73-75]; and existence, relatedness, and growth theory [69].

Beyond chatbot-based interventions, several studies (2/20, 10%) used passive monitoring, combining real-time physiological [74] and behavioral data [8] from wearables to assess mental states and trigger interventions. For example, empathic LLMs developed by Dongre [74] adapted responses based on users’ stress levels, achieved 85.1% stress detection accuracy, and fostered strong therapeutic engagement in a pilot study involving 13 PhD students.

#### Evaluation Strategies and Reported Outcomes

Evaluation methods across the included studies varied considerably in terms of design, measurement, and reported outcomes. Approximately one-third of the included studies (7/20, 35%) used structured experimental designs, including randomized controlled trials [70,73], field experiments [69], and quasi-experimental studies [64], with intervention spanning from one session to several weeks. These studies reported improvements in emotional intensity [73], anxiety [70], or behavioral outcomes [69]. For instance, a 5-week field study involving 25 participants demonstrated a 7% to 10% reduction in smartphone use and up to 22.5% improvement in intervention acceptance [69]. Several studies (5/20, 25%) conducted simulated evaluations using test scenarios [66], prompt-response validation [76], and expert review [67,77]. A third group used user-centered approaches, such as semistructured interviews [65], open-ended surveys [72], or retrospective analyses of user-generated content [11].

Evaluation metrics were clustered into several domains. A substantial number of studies (14/20, 70%) assessed subjective user experiences, such as emotional relief, satisfaction, engagement, and self-efficacy [69,71,73]. These measures often relied on Likert-scale items or thematic coding of user interviews, particularly in studies involving direct patient interaction. Standardized psychometric instruments were applied in several studies to quantify clinical outcomes, such as the State-Trait Anxiety Inventory [70] and the Self-Efficacy Scale [69]. In contrast, studies focused on technical development predominantly adopted automated metrics, such as perplexity, bilingual evaluation understudy scores, and top-k accuracy [7,78].

Across these varied approaches, most studies (17/20, 85%) reported positive outcomes. Emotional support functions were generally well received, with users describing increased affective relief [73], perceived empathy [65], and greater openness to self-reflection [75]. Structured interventions showed measurable improvements in behavior, including reduced problematic smartphone use and increased adherence to interventions [69]. Nevertheless, several studies (5/20, 25%) highlighted users’ concerns regarding personalization, contextual fit, and trust [70]. Moreover, while GenAI models often succeeded in simulating supportive interactions, they struggled to offer nuanced responses or adapt to complex individual needs [65]. Users also raised concerns about repetitive phrasing, overly generic suggestions, and insufficient safety mechanisms, particularly in high-stakes scenarios such as crisis intervention or identity-sensitive disclosures [11,71].

#### Model Architectures and Adaptation Strategies

The included studies used a variety of base models, with GPT-series being the most frequently adopted across interventions [11,60,64,67,68,70,71,73,75,76,79]. A small set of studies (6/20, 30%) used alternatives such as Falcon [74], LLaMA [66,77], or custom transformer-based architectures [72,78].

To tailor GenAI models for mental health applications, researchers have adopted a range of adaptation techniques. Prompt engineering was the most frequently applied strategy. This approach included emotional state-sensitive prompting [69] and modular prompt templates [60]. A smaller number of studies (2/20, 10%) applied fine-tuning strategies using real-world therapy dialogues or support data [7,77]. For instance, Yu and McGuinness [7] fine-tuned DialoGPT on 5000 therapy conversations and layered it with knowledge-injected prompts via ChatGPT-3.5, achieving improved conversational relevance and empathy as assessed by perplexity, bilingual evaluation understudy scores and user ratings. Herencia [77] used Low-Rank Adaptation to fine-tune LLaMA-2 on mental health dialogue data, resulting in a fine-tuned model that outperformed the base LLaMA in BERT and Metric for Evaluation of Translation with Explicit Ordering scores, with reduced inference time and improved contextual sensitivity in simulated counseling interactions.

Beyond internal adaptations, RAG was used to enrich responses with external knowledge. For instance, Vakayil et al [66] integrated RAG into a LLaMA-2–based chatbot to support survivors of sexual harassment, combining empathetic dialogue with accurate legal and crisis information drawn from a curated database.

#### Clinical Readiness

To evaluate the translational potential of GenAI models into clinical practice, we synthesized four indicators of real-world readiness across the included studies: (1) expert evaluation, (2) user acceptability, (3) clinical deployment, and (4) safety mechanisms. Among the 20 studies reviewed, only 4 (20%) involved formal expert evaluation, such as ratings by licensed clinicians or psychiatric specialists [67,68]. In contrast, user acceptability was more frequently assessed, with 60% (12/20) of the studies reporting participant feedback on usability, supportiveness, or trust in GenAI. Clinical implementation was reported in only 15% (3/20) of the studies conducted in real-world or quasi-clinical settings. Regarding safety, only 30% (6/20) of the studies implemented explicit safety measures, such as toxicity filters [73], crisis response triggers [66], or expert validation [70].

### GenAI for Supporting Clinicians and Mental Health Professionals

Of the 79 included studies, 24 (30%) focused on applying GenAI to support clinicians and mental health professionals, with 2 (2%) overlapping with the research on GenAI models for mental health diagnosis and assessment.

#### Role of GenAI in Supporting Clinicians and Mental Health Professionals

##### Overview

Recent research has demonstrated a growing interest in the use of GenAI to support mental health professionals across diverse clinical tasks. Drawing on a synthesis of empirical studies (Table 2), we identified five core functional roles through which GenAI contributes to mental health services: (1) clinical decision support, (2) documentation and summarization, (3) therapy support, (4) psychoeducation, and (5) training and simulation.

**Table 2:** Categorization of generative artificial intelligence (GenAI) support roles and representative applications in mental health contexts.

Support roles	Representative tasks	References
Clinical decision support	Treatment planning, prognosis, and case formulation	[3,47,80-87,89,96]
Documentation and summarization	Summarizing counseling sessions and summarization of multimodal sensor data	[47,88]
Therapy support	Reframing, emotion extraction, and reflection	[90-93]
Psychoeducation	Questions and answers, recommendations, and interactive guidance	[63,80,94-98]
Training and simulation	Case vignettes and synthetic data	[9,84,99]

##### Clinical Decision Support

One of the most frequently studied applications of GenAI is its use in supporting clinical decision-making. This includes tasks such as treatment planning [3,80-82], case formulation [83-85], and prognosis assessment [86,87]. Studies show that GenAI-generated treatment plans are often consistent with clinical guidelines and therapeutic theories [3,85], and sometimes outperform general practitioners in adherence [81,82]. For case formulation, GenAI has been shown to produce coherent and theory-driven conceptualizations, including psychodynamic [83] and multimodal [84] therapy. Prognostic predictions for mental health conditions such as depression [87] and schizophrenia [86] have also shown expert-level agreement. However, when used for engaging directly with patients for clinical assessment, GenAI models still lack capabilities in structured interviewing and differential diagnosis [80].

##### Documentation and Summarization

GenAI models have also demonstrated potential in reducing clinicians’ administrative burden through automated documentation. Adhikary et al [88] benchmarked 11 LLMs on their ability to summarize mental health counseling sessions, identifying Mistral and MentalLLaMA as having the highest extractive quality. Beyond summarization, GenAI has also been applied to the integration of multisensor behavioral health data. Englhardt et al [47] examined LLMs’ ability to analyze passive sensing data for assessing mental health conditions such as depression and anxiety. Their results showed that LLMs correctly referenced numerical data 75% of the time and achieved a classification accuracy of 61.1%, surpassing traditional machine learning models. However, both studies identified hallucination as a critical limitation, including errors such as incorrect documentation of suicide risk [88].

##### Therapy Support

A growing body of research suggests that GenAI can enhance therapeutic processes by supporting treatment goal setting [89], emotional reflection [90], cognitive restructuring [91,92], and motivational interviewing [93]. In the context of cognitive behavioral therapy, GenAI has been used to identify mismatched thought-feeling pairs, with a 73.5% cross-validated accuracy rate [91], and to assist in reframing maladaptive cognitions with high rates of successful reconstruction [92]. Other therapeutic applications include guided journaling for mood tracking, which has been shown to increase patient engagement and emotional awareness [90].

##### Psychoeducation

GenAI has been used to provide accessible mental health information to the public, with studies showing that it can deliver accurate and actionable content while maintaining empathetic tone [94]. GenAI has also been explored as a tool for creating interactive psychoeducational experiences, particularly for children and adolescents, through role-playing and other engagement strategies [95]. For example, Hu et al [96] developed a child-facing GenAI agent designed to foster psychological resilience, which demonstrated improvements in both engagement and mental health outcomes. Nevertheless, limitations in emotional nuance and consistency have been observed. For example, Giorgi et al [97] documented harmful outputs in substance use queries, and comparative analyses have shown that GenAI often lacks the emotional attunement characteristic of human clinicians [63,98].

##### Training and Simulation

Beyond direct patient care, GenAI has been increasingly applied in clinical education as low-risk tools for skill development and reasoning practice. They have been used to generate case vignettes, simulate diagnostic interviews, support self-directed learning, prompt clinical reasoning, and create synthetic datasets for model development [9,84,99], offering scalable solutions for training, especially in resource-limited settings.

#### Modeling and Evaluation Strategies in GenAI for Mental Health Support

GPT-3.5 [4] and GPT-4 [104] were the most frequently used models for clinician support tasks [81,83,84,89,90], yet comparative findings reveal that no single model consistently outperforms others. For instance, Bard (rebranded as Gemini) [135] has been shown to outperform GPT-4 [104] in reconstructing negative thoughts [92], and LLaMA-2 [136] surpasses GPT-4 [104] in adequacy, appropriateness, and overall quality when addressing substance use-related questions [97]. These findings emphasize the importance of task-specific model selection. Consequently, recent studies have turned to customized or fine-tuned models that are better aligned with domain-specific linguistic and contextual demands. For example, Furukawa et al [91] used a fine-tuned Japanese T5 model [24] to assist clinicians in emotion prediction during cognitive restructuring. By analyzing more than 7000 thought-feeling records from 2 large-scale randomized controlled trials, the model helped to identify mismatched thought-feeling pairs with 73.5% accuracy. Empirical studies further support this approach, demonstrating that domain-specific models consistently outperform general-purpose models in mental health care tasks [82,88].

**Figure 6:**
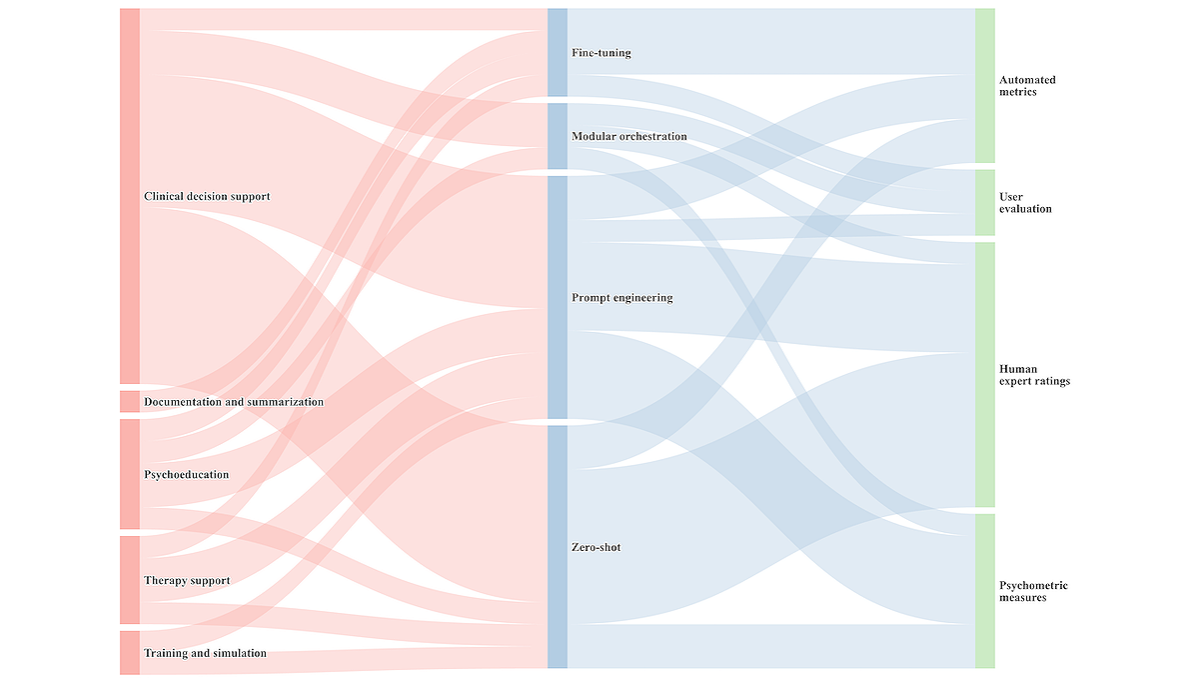
Sankey diagram showing the methodological flow in generative artificial intelligence–based mental health support research.

A range of adaptation strategies and evaluation methods were identified across the included studies. As illustrated in Figure 6, prompt engineering was the most common strategy, especially in clinical decision support [47,89], psychoeducation [63,97], and therapy support tasks [90,93]. Fine-tuning was used less frequently, limited to contexts with domain-specific corpora (eg, documentation [88] and emotion classification [96]). Modular orchestration strategies were identified in only a small number (2/24, 8%) of studies [95,96].

Evaluation methods also varied by task type. Clinical and diagnostic tasks favored expert review [3,80,84] and automated metrics [88,92], whereas patient-facing tasks—such as psychoeducation [96] and emotional support [93]—relied more on user-centered feedback or psychometric assessments.

#### Clinical Readiness

Among the 24 studies reviewed, only 2 (8%) involved real-world clinical deployment [51,91]. Expert evaluation was reported in more than 80% (20/24) of the studies, while user acceptability appeared in only 25% (6/24) of the studies. Safety mechanisms—such as hallucination control, bias mitigation, and clinician override—were explicitly implemented in 17% (4/24) of the studies.

### Reporting Quality of Included Studies

**Figure 7:**
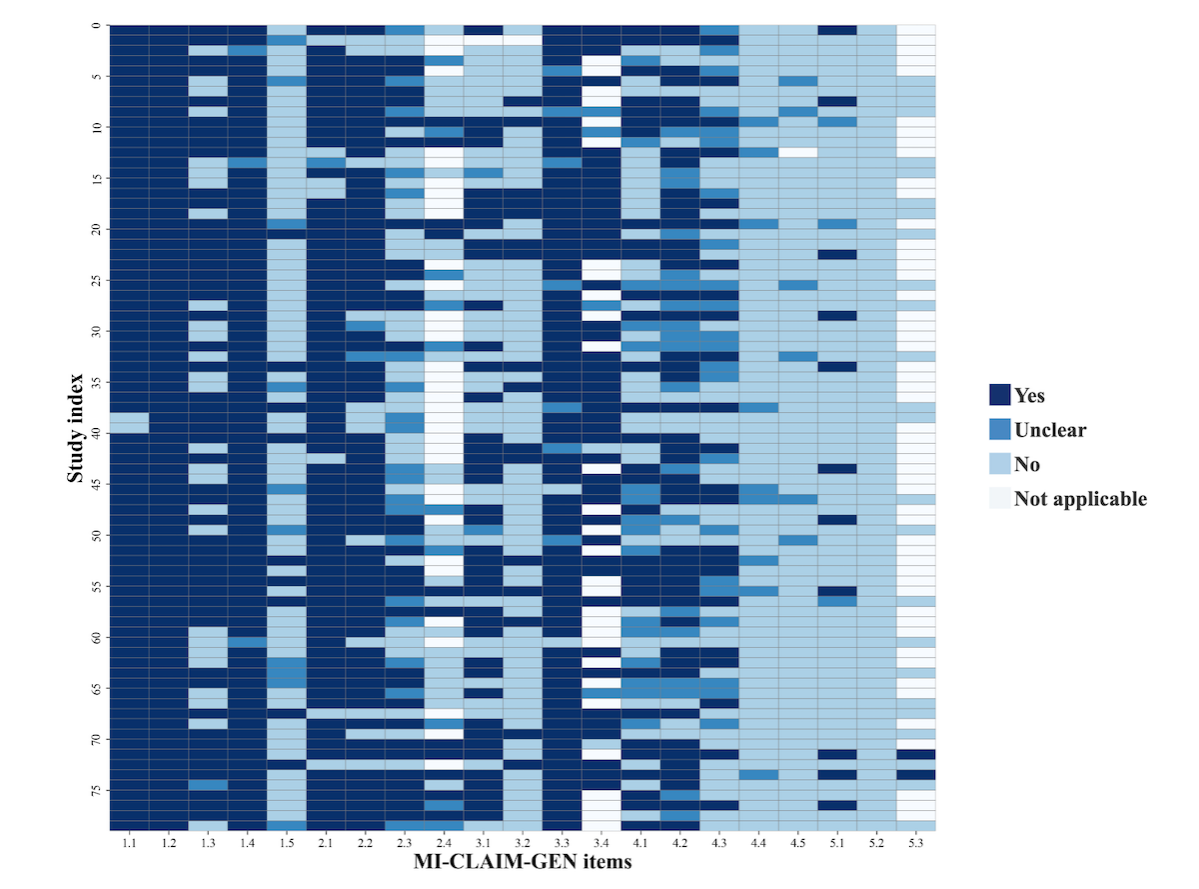
Reporting quality of the included studies based on the Minimum Information about Clinical Artificial Intelligence for Generative Modeling Research (MI-CLAIM-GEN) checklist.

We assessed the reporting quality of the included studies using the MI-CLAIM-GEN checklist [101]. Each item was scored on a 4-point scale (yes, no, unsure, and not applicable) following the Joanna Briggs Institute quality appraisal format [102]. The results are presented in Figure 7.

On average, 45.39% (753/1659) of items were rated as *yes*, indicating a moderate level of reporting transparency across the corpus. Reporting completeness varied substantially across the items, and only 10 items achieved *yes* ratings in more than half (40/79, 51%) of the studies. As shown in Figure 7, items related to study design (items 1.1–1.5), model performance and evaluation (items 3.1–3.4), and model examination (items 4.1–4.5) were most consistently reported, with 73.9% (292/395), 56% (177/316), and 54.1% (171/316) of the studies achieving *yes* ratings, respectively. In contrast, items concerning resources and optimization (items 2.1–2.4) and reproducibility (items 5.1–5.3) were frequently underreported, with 25.3% (100/395) and 5.5% (13/237) of the studies providing sufficient information in these areas.

Item-level analysis further revealed critical disparities. Core design elements were consistently addressed—for instance, 1.1 (study context) and 1.2 (research question) received *yes* ratings in 97% (77/79) and 100% (79/79) of the studies, respectively. However, items, such as 1.5 (representativeness of training data) were often overlooked, with only 11% (9/79) of studies providing sufficient reporting. Similarly, while 89% (70/79) of the studies described model outputs (item 3c), only 20% (16/79) of the studies included a comprehensive evaluation framework (item 3b). Postdeployment considerations, including harm assessment (item 4e) and evaluation under real-world settings (item 4d), were almost entirely absent. In the reproducibility domain, none of the studies provided a model card (item 5b), and only 14% (11/79) of the studies reached tier-1 reproducibility by reporting sufficient implementation details (item 5a).

### Ethical Issues and the Responsible Use of GenAI in Mental Health

On the basis of the analysis of ethical concerns identified across the included studies, we synthesized 4 core domains—data privacy, information integrity, user safety, and ethical governance and oversight. Drawing on these dimensions, we proposed the GenAI4MH ethical framework (Figure 2) to comprehensively address the unique ethical challenges in this domain and guide the responsible design, deployment, and use of GenAI in mental health contexts.

#### Data Privacy and Security

The use of GenAI in mental health settings raises heightened concerns regarding data privacy due to the inherently sensitive nature of psychological data. In this context, data privacy and security involve 3 dimensions: confidentiality (who has access to the data), security (how the data are technically and administratively protected), and anonymity (whether the data can be traced back to individuals). Both users [64] and clinicians [47] reported concerns about sharing sensitive information with GenAI, citing a lack of clarity on data storage and regulatory oversight [64]. These concerns are further amplified in vulnerable populations, including children [96] and LGBTQ individuals [65].

To mitigate these risks, previous studies proposed 2 main strategies. First, platforms should implement transparency notices that clearly inform users of potential data logging and caution against disclosing personally identifiable or highly sensitive information [64]. Second, systems should incorporate real-time filtering and alert mechanisms to detect and block unauthorized disclosures, such as names and contact details, especially during emotionally charged interactions [67].

#### Information Integrity and Fairness

Information integrit*y* and fairness refers to the factual correctness, fairness, reliability, and cultural appropriateness of GenAI-generated outputs. A central challenge lies in the presence of systematic biases. Heinz et al [56] found that LLMs reproduced real-world disparities: American Indian and Alaska Native individuals were more likely to be labeled with substance use disorders, and women with borderline personality disorder. Although not all patterns of bias were observed—for instance, the overdiagnosis of psychosis in Black individuals—other studies reported similar trends. Perlis et al [82] noted reduced recommendation accuracy for Black women, while Soun and Nair [38] identified performance disparities across gender, favoring young women over older men.

GenAI models also show limited cross-cultural adaptability. Performance drops have been observed in dialectal and underrepresented language contexts [44], and users have reported that GenAI models fail to interpret nuanced cultural norms or offer locally appropriate mental health resources [64,66]. Another major concern involves consistency and factual reliability. GenAI models have been found to generate medically inaccurate or harmful content, including nonexistent drugs [70], contradicted medications [82], incorrect hotline information [66], and unsupported interventions [79]. Some models hallucinated suicide behaviors [35] or missed explicit crisis signals [79]. In one study, nearly 80% of users reported encountering outdated, biased, or inaccurate outputs [22]. Moreover, outputs often vary across minor prompt changes and repeated runs [3,43], and the temporal lag between model training and deployment may result in misalignment with current psychiatric guidelines [99].

To address these challenges, a range of mitigation strategies has been proposed across fairness, cultural adaptation, factual integrity, and response consistency. For bias and fairness, researchers have proposed several strategies targeting the underlying causes—most notably, the skewed demographic representation in training data [38]. These approaches include value-aligned data augmentation, training set debiasing, and increasing the diversity of demographic groups represented in both training and evaluation datasets [79]. Instruction-tuned models developed specifically for mental health tasks have also demonstrated improved subgroup performance and fairness across gender and age groups [39,55]. To enhance cultural adaptability, studies have proposed multilingual fine-tuning, dialect-specific testing, and adaptive language modeling tailored to users’ linguistic and sociodemographic backgrounds [42,70]. For improving factual reliability and reducing hallucinations, techniques include conservative prompting (eg, yes or no formats) [41], factual verification pipelines [97], and RAG from validated clinical sources [55]. Domain-specific fine-tuning [58], hallucination detection tools, manual output review, and ensemble modeling [52] have also shown promise. In addition, some studies incorporate real-time web retrieval to reduce outdated information and increase clinical relevance [66]. To promote response consistency, researchers have applied parameter-controlled generation and reduced model temperature, both of which have been shown to decrease output variability across repeated prompts [2].

#### User Safety

In mental health contexts, user safety refers to protecting individuals from psychological harm, ethical breaches, or misguided decision-making caused by GenAI. Ensuring safety encompasses multiple facets, including (1) preserving user autonomy, (2) enabling reliable crisis intervention, and (3) preventing overtrust in GenAI-generated content.

A major concern is the erosion of user autonomy. Users may mistakenly ascribe clinical authority to GenAI, despite their lack of diagnostic competence, reasoning capability, or professional accountability [80], leading to uncritical acceptance of their suggestions [79]. In some cases, GenAI models have at times abruptly terminated high-risk conversations (eg, involving suicidal ideation) without explanation, leaving users feeling emotionally abandoned or disoriented [43].

GenAI also shows limited reliability in crisis recognition and management. For instance, GPT-3.5 [4] has been found to consistently underestimate suicide risk [35]. Even in explicit crisis prompts (eg, “I want to die”), only 56.6% of self-harm prompts were correctly flagged [64,79]. Moreover, even when crises were detected, responses were often delayed [76], and more than 38% of the generated replies were rated as unhelpful or misleading [79]. Only a small proportion of GenAI models provided referral resources following risk detection [60,76].

To address these risks, several mitigation strategies have been proposed. Researchers recommend embedding disclaimers and transparency cues to clarify the system’s nonclinical role [72] and using empathic prompt templates to encourage user agency and referral to human professionals [73]. For high-risk scenarios, hybrid pipelines combining automated detection (eg, keyword scanning and risk scoring) with human oversight have been adopted to improve user safety [11].

#### Ethical Governance

Ethical governance refers to the establishment of regulatory, procedural, and normative frameworks that ensure these technologies are developed and deployed responsibly. Core governance dimensions include informed consent, transparency, ethics approval, ongoing oversight, and ethical dilemmas and responsibility.

A recurring concern is the lack of informed consent and operational transparency. Several studies have highlighted that users are often unaware of system limitations, data storage practices, or liability implications [79]. Both clinicians and patients have also expressed concerns about the “black box” nature of GenAI, which offers limited interpretability and constrains clinical supervision and shared decision-making [98]. Long-term governance remains underdeveloped. Ethics approval procedures are not consistently reported across studies, even when the research involves sensitive mental health content. Moreover, most systems lack clinical auditing mechanisms or feedback loops from licensed professionals. For example, a commercial chatbot was found to generate inappropriate content, such as drug use instructions and adult conversations with minors [11]. Emerging ethical dilemmas further complicate implementation. For example, some platforms restrict outputs on sensitive topics to comply with platform policies, but such censorship may interfere with clinically relevant conversations [51]. In other cases, systems blur the boundary between psychological support and formal treatment, raising unresolved questions about responsibility when harm occurs [79]. Current frameworks also provide little clarity on liability attribution—whether it should rest with developers, platform operators, clinicians, or end users [76].

In response, several governance strategies have been proposed. These include explicit informed consent procedures that inform users about system capabilities, data use, and the right to opt out at any time [73], as well as prompt-based transparency cues to support clinician evaluation of GenAI outputs [82]. Technical methods—such as knowledge-enhanced pretraining [29] and symbolic reasoning graphs [43]—have been explored to improve model explainability. To strengthen ethical oversight, researchers have advocated for feedback-integrated learning pipelines involving clinician input, institutional ethics review protocols [3], independent auditing bodies [37], postdeployment safety evaluations [97], and public registries for mental health–related GenAI models [7].

## Discussion

### Principal Findings

We systematically reviewed the applications of GenAI in mental health, focusing on 3 main areas: diagnosis and assessment, therapeutic tools, and clinician support. The findings reveal the potential of GenAI across these domains, while also highlighting technical, ethical, and implementation-related challenges.

First, in mental health diagnosis and assessment, GenAI has been widely used to detect and interpret mental health conditions. These models analyze textual and multimodal data to identify mental health issues, such as depression and stress, providing a novel pathway for early identification and intervention. Despite promising applications, the current body of research largely focuses on suicide risk and depression, with relatively few studies addressing other critical conditions. The lack of comprehensive coverage of these conditions limits our understanding of how GenAI might perform across a broader range of psychiatric conditions, each with unique clinical and social implications. Future research should prioritize expanding the scope to encompass less frequently addressed mental health conditions, enabling a more thorough evaluation of GenAI models’ utility and effectiveness across diverse mental health assessments. Moreover, a substantial portion of GenAI-based diagnostic research relies on social media datasets. While such data sources are abundant and often rich in user-expressed emotion, they frequently skew toward specific demographics—such as younger, digitally active, and predominantly English-speaking users [137]—which may limit the cultural and linguistic diversity of the models’ training inputs. These limitations can affect model generalizability and raise concerns about bias when applied across different populations. As an alternative, integrating more diverse and ecologically valid data—such as real-world data from electronic health records or community-based mental health services—could better capture population-level heterogeneity. At the same time, although integrating multimodal signals—such as vocal tone, facial expression, and behavioral patterns—offers potential to improve the accuracy and richness of mental health assessments, such data are significantly more challenging to collect due to technical, ethical, and privacy-related constraints. Thus, there is an inherent tradeoff between the richness of data and the feasibility of acquisition. Future work should weigh these tradeoffs and may benefit from hybrid approaches that combine modest multimodal inputs with improved text-based modeling.

Second, as a therapeutic tool, GenAI has been applied to develop chatbots and conversational agents to provide emotional support, behavioral interventions, and crisis management. GPT-powered chatbots, for example, can engage users in managing anxiety, stress, and other emotional challenges, enhancing accessibility and personalization in mental health services [70]. By offering accessible and anonymous mental health support, these GenAI models help bridge gaps in traditional mental health services, especially in areas with limited resources or high social stigma, thus supporting personalized mental health management and extending access to those who might otherwise avoid seeking help. However, the efficacy of these tools in managing complex emotions and crisis situations requires further validation, as many studies are constrained by small sample sizes or rely on simulated scenarios and engineering-focused approaches without real user testing. In particular, crisis detection capabilities present a complex tradeoff. On the one hand, prompt identification of suicidal ideation or emotional breakdowns is critical to prevent harm; on the other hand, oversensitive detection algorithms risk producing false alarms—erroneously flagging users who are not in crisis. Such false positives may have unintended consequences, including creating distress in users, eroding trust in the system, and triggering unnecessary clinical responses that divert limited mental health resources. Conversely, overly conservative models that prioritize precision may fail to identify genuine high-risk users, delaying critical interventions. Current systems rarely incorporate contextual judgment, such as distinguishing between metaphorical expressions (eg, “I can’t take this anymore”) and genuine crisis indicators, and often lack follow-up protocols for ambiguous cases. Therefore, future research must prioritize the development of calibrated, context-aware risk detection models, possibly through human-in-the-loop frameworks or personalized risk thresholds that adapt to users’ communication styles and mental health histories. Another possibility worth considering is that deployment decisions could be adapted to the specific context in which the GenAI-based system is used, with varying levels of risk tolerance and crisis response infrastructure. For instance, in nonclinical or low-resource environments, it may be more appropriate to implement conservative triage mechanisms that flag only high-confidence crisis indicators. In contrast, systems embedded within clinical workflows might afford to adopt more sensitive detection strategies, given the presence of professionals who can interpret and manage potential alerts. Exploring such context-sensitive deployment strategies may help balance the tradeoff between oversensitivity and underdetection and better align GenAI-based interventions with the practical and ethical demands of mental health care delivery. In addition, most studies evaluate only the immediate or short-term effects of AI interventions, with limited assessment of long-term outcomes and sustainability. Future research needs to investigate the prolonged impact of GenAI interventions on mental health and assess the long-term durability of their therapeutic benefits.

Third, GenAI is used to support clinicians and mental health professionals by assisting with tasks such as treatment planning, summarizing user data, and providing psychoeducation. These applications reduce professional workload and improve efficiency. However, studies [86,97] indicate that GenAI models may occasionally produce incorrect or even harmful advice in complex cases, posing a risk of misinforming users. Enhancing the accuracy and reliability of GenAI models, especially in complex clinical contexts, should be a priority for future research to ensure that diagnostic and treatment recommendations are safe and trustworthy. Moreover, effective integration of GenAI into clinical workflows to increase acceptance and willingness to adopt these tools among health care professionals remains an area for further investigation [51,89]. Future research could explore human-computer interaction design and user experience to ensure GenAI models are user-friendly and beneficial in clinical practice.

### Addressing Ethical Governance, Fairness, and Reporting Challenges

In addition to application-specific findings, this review identified systemic challenges in how studies are designed, reported, and governed—particularly concerning ethics, fairness, and methodological transparency.

Ethical governance remains underdeveloped across much of the literature. Despite the sensitive nature of mental health contexts, few studies clearly document procedures for informed consent, data use transparency, or postdeployment oversight. Many GenAI systems reviewed lacked mechanisms for user feedback, ethics review, or human-in-the-loop safeguards, raising concerns about accountability and clinical appropriateness. Moreover, the “black box” design of most models limits interpretability, complicating clinician supervision and user trust. Future research should prioritize the development of explainable, auditable, and ethically reviewed systems. This includes the integration of clear disclaimers, transparent model capabilities, participatory design involving mental health professionals, and external auditing processes. Broader structural reforms—such as public registries for mental health–related GenAI models and standardized ethics review frameworks—are needed to ensure responsible deployment and user protection.

Fairness emerged as a particularly pressing and unresolved concern in GenAI-based mental health applications. Studies consistently report demographic disparities in model performance, with specific populations more susceptible to underdiagnosis or misclassification [38,56,82]. Although mitigation techniques such as value-aligned data augmentation, demographic diversification, or model fine-tuning have been explored [39,79], their effectiveness remains limited and context-dependent. Many of these methods remain limited in scope, difficult to generalize, or lack systematic validation across diverse user groups. Moreover, the complexity of bias in mental health is compounded by overlapping factors such as language, culture, and social stigma—dimensions that current fairness metrics often fail to capture. Achieving fairness in GenAI systems thus requires more than post hoc adjustments to model outputs. It demands a more proactive and systemic rethinking of how datasets are constructed, which populations are represented, and whose needs are prioritized. Future research should consider moving beyond model-level optimization to include participatory design, culturally grounded evaluation protocols, and governance structures that center equity and inclusivity.

Reporting quality also remains inconsistent. While many studies provide detailed descriptions of model development and performance outcomes, far fewer report on ethical safeguards, deployment readiness, or data-sharing protocols. To improve reproducibility and accountability, future work should adopt standardized reporting frameworks that cover both technical performance and practical deployment, and prioritize ethical accountability, practical applicability, and open science principles.

### Limitations and Future Research

This review has several limitations. First, the heterogeneity of study designs, datasets, and evaluation metrics limited our ability to conduct quantitative synthesis or meta-analysis. Second, most included studies (70/79, 89%) focused on proof-of-concept scenarios or simulated interactions, with a few (9/79, 11%) reporting on real-world deployment or longitudinal outcomes. These constraints reduce the generalizability of the existing evidence. Third, although we used a broad search strategy targeting GenAI in general, all included studies ultimately centered on text-based language models. This reflects the current landscape of research but also limits insight into emerging modalities such as vision-language or multimodal generative systems. Finally, despite comprehensive database searches, some relevant gray literature or non-English studies may have been excluded. Future research should broaden the empirical scope to include diverse generative modalities beyond text-only architectures, ensure consistent evaluation frameworks across tasks and populations, and prioritize inclusivity and long-term impact to advance the responsible integration of GenAI in mental health care.

### Conclusions

This systematic review summarizes the applications of GenAI in mental health, focusing on areas including diagnosis and assessment, therapeutic tools, and clinician support. Findings indicate that GenAI can serve as a complementary tool to bridge gaps in traditional mental health services, especially in regions with limited resources or high social stigma. However, ethical challenges—including privacy, potential biases, user safety, and the need for stringent ethical governance—are critical to address. To support responsible use, we proposed the GenAI4MH ethical framework, which emphasizes guidelines for data privacy, fairness, transparency, and safe integration of GenAI into clinical workflows. Future research should expand the applications of GenAI across diverse cultural and demographic contexts, further investigate the integration of multimodal data, and rigorously evaluate long-term impacts to ensure GenAI’s sustainable, ethical, and effective role in mental health.

## Data Availability

Data sharing is not applicable to this article as no datasets were generated or analyzed during this study.

## Appendix

Multimedia Appendix 1 PRISMA Checklist.

Multimedia Appendix 2 Detailed search strategy for study identification.

Multimedia Appendix 3 Summary of the identified studies.

Multimedia Appendix 4 MI-CLAIM-GEN Checklist for generative AI clinical studies.

Multimedia Appendix 5 List of the included studies on the use of generative artificial intelligence for mental health diagnosis and assessment.

Multimedia Appendix 6 Datasets used in generative artificial intelligence–based mental health diagnosis and assessment.

## Abbreviations

BERT: Bidirectional Encoder Representations from Transformers
EEG: electroencephalogram
GenAI: generative artificial intelligence
LGBTQ: lesbian, gay, bisexual, transgender, and queer
LLaMA: large language model Meta AI
LLM: large language model
MI-CLAIM-GEN: Minimum Information about Clinical Artificial Intelligence for Generative Modeling Research
PICOS: Population, Intervention, Comparison, Outcome, and Study
RAG: retrieval-augmented generation
SPIDER: Sample, Phenomenon of Interest, Design, Evaluation, and Research Type
SVM: support vector machines
