# Supplementary material for: The Application and Ethical Implication of Generative AI in Mental Health: Systematic Review
Source: JMIR Ment Health. 2025 Jun 27;12:e70610. doi: 10.2196/70610 (PMC12254713; doi:10.2196/70610)
Supplement: Multimedia Appendix 2 [file mental_v12i1e70610_app2.pdf]

Supplementary Table 1. Search Strategies

Source	Search strategy	Hits retrieved
1. EMBASE<br>1980- (Ovid SP)	1. mental health.ab,ti<br>2. psychiatry.ab,ti<br>3. psychology.ab,ti<br>4. mental disorder.ab,ti<br>5. depression.ab,ti<br>6. anxiety.ab,ti<br>7. 1 or 2 or 3 or 4 or 5 or 6<br>8. Generative AI.ab,ti<br>9. Large Language Model.ab,ti<br>10. chatgpt.ab,ti<br>11. 8 or 9 or 10<br>12. 7 and 11	229
2. PSYCINFO<br>1806- (Ovid SP)	1. mental health.TI<br>2. psychiatry.TI<br>3. psychology.TI<br>4. mental disorder.TI<br>5. depression.TI<br>6. anxiety.TI<br>7. 1 or 2 or 3 or 4 or 5 or 6<br>8. Generative AI.TI<br>9. Large Language Model.TI<br>10. chatgpt.TI<br>11. 8 or 9 or 10<br>12. 7 and 11	72
3. PUBMED	1. "mental health"[Title/Abstract]<br>2. "mental disorder"[Title/Abstract]<br>3. psychiatry[Title/Abstract]<br>4. psychology[Title/Abstract]<br>5. depression[Title/Abstract]<br>6. anxiety[Title/Abstract]<br>7. 1 or 2 or 3 or 4 or 5 or 6<br>8. "Generative AI"[Title/Abstract]<br>9. "Large Language Model"[Title/Abstract]<br>10. chatGPT[Title/Abstract]<br>11. 8 or 9 or 10<br>12. 7 and 11	204
4. SCOPUS	1. TITLE-ABS-KEY("mental health")<br>2. TITLE-ABS-KEY("mental disorder")<br>3. TITLE-ABS-KEY("psychiatry")<br>4. TITLE-ABS-KEY("psychology")<br>5. TITLE-ABS-KEY("depression")<br>6. TITLE-ABS-KEY("anxiety")<br>7. 1 or 2 or 3 or 4 or 5 or 6<br>8. TITLE-ABS-KEY("Generative AI")<br>9. TITLE-ABS-KEY("Large Language Model")<br>10. TITLE-ABS-KEY("ChatGPT")<br>11. 8 or 9 or 10<br>12. 7 and 11	36
5. ACM Digital Library	1. mental health.ab<br>2. psychiatry.ab<br>3. psychology.ab<br>4. mental disorder.ab<br>5. depression.ab<br>6. anxiety.ab<br>7. 1 or 2 or 3 or 4 or 5 or 6<br>8. Generative AI.ab	143

	9. Large Language Model.ab<br>10. chatgpt.ab<br>11. 8 or 9 or 10<br>12. 7 and 11	
6. Google Scholar	1. mental health.<br>2. psychiatry.<br>3. psychology.<br>4. mental disorder.<br>5. depression.<br>6. anxiety.<br>7. 1 or 2 or 3 or 4 or 5 or 6<br>8. Generative AI.<br>9. Large Language Model.<br>10. chatgpt.<br>11. 8 or 9 or 10<br>12. 7 and 11	99
TOTAL before de-duplication		783
TOTAL after de-dupe and first-assess		228
