# Supplementary material for: The Application and Ethical Implication of Generative AI in Mental Health: Systematic Review
Source: JMIR Ment Health. 2025 Jun 27;12:e70610. doi: 10.2196/70610 (PMC12254713; doi:10.2196/70610)
Supplement: Multimedia Appendix 3 [file mental_v12i1e70610_app3.pdf]

Supplementary Table 2. Data extraction summary.

Study	Study Index	Sample (S)	Phenomenon of Interest / Intervention (PI/I)	Design (D)	Evaluation / Outcome (E/O)	Research Type (R)	Comparator (C)
Adhikary et al. (2024)	1	191 counseling sessions, including 11,543 utterances; dyadic therapist–patient dialogues from publicly available sources (e.g., YouTube); annotated with 3 components: symptom/history, patient discovery, reflection	Counseling-component–guided summarization using 11 LLMs (e.g., MentalLlama, Mistral, MentalBART)	Benchmarking experiment using real-world annotated data (MentalCLOUDS); model-based evaluation	Quantitative: ROUGE-1/2/L, BERTScore; Qualitative: expert ratings on 6 parameters (e.g., coherence, ethicality, perceived effectiveness), hallucination level	Quantitative	Compared 11 LLMs (MentalLlama, Mistral, MentalBART, BART, GPT-J, etc.)

Alanezi (2024)	2	24 outpatients (16 males, 8 females) from a Saudi hospital; aged 18+; with anxiety, depression, or behavioral disorders	Use of GPT-3.5 for delivering mental health support	Quasi-experimental + semi-structured qualitative interviews	Thematic analysis of interview data: Eight positive themes (e.g., psychoeducation, emotional support, CBT techniques), four negative themes (e.g., accuracy concerns, cultural limitations). Data collected via interviews (~54 min each), coded with NVivo.	Qualitative	N/A
Alessa & Al-Khalifa (2023)	3	Elderly personas (5 different virtual profiles based on age, health, interests, etc.); human evaluators (1 professor, 1 graduate student)	Use of GPT-3.5-based conversational companion to alleviate loneliness and social isolation among the elderly	System design and simulated qualitative evaluation	Human evaluation (7 criteria: engagingness, interestingness, inquisitiveness, listening, avoiding repetition, fluency, making sense) on conversations with simulated elderly personas	Qualitative	N/A

AlHamed et al. (2024)	4	Reddit users in the UMD Suicidality Dataset v2; post-level and user-level texts from r/SuicideWatch, annotated with suicide risk levels (Low, Moderate, High)	Use of Meta Llama 2 7B to extract textual evidence (summaries and highlights) supporting pre-assigned suicide risk levels	Systematic evaluation on shared task benchmark; zero-shot prompting with LLMs; no clinical experiment or user testing	Summary Consistency (0.964), Contradiction (0.060); Highlight Precision (0.899), Harmonic Mean (0.888), Recall (0.577); Qualitative and quantitative evaluation using NLP metrics (e.g., BERTScore)	Quantitative	None
Bauer et al. (2024)	5	Reddit users (N ≈ 2.9 million posts from 30 subreddits), including r/SuicideWatch and other mental health/non–mental health communities	Suicidality-related discourse on Reddit; linguistic expression of suicide-related mental states	Large-scale computational analysis using LLMs (BERT, GPT-4), dimensionality reduction, XAI	Use of BERT embeddings, SVD, ProtoDash, and GPT-4 to extract latent linguistic dimensions; outcomes include emotional well-being, support-seeking behavior, and severity of distress; mapping to suicide theories (ITS, 3ST, IMV) and HiTOP model	Qualitative	N/A

Berrezueta-Guzman et al. (2024)	6	Ten experts in child ADHD therapy; therapists interacted with custom GPT-3.5	Use of a custom GPT-3.5 to enhance ADHD therapy through AI-mediated interaction	Delphi method with expert validation in a simulated therapy setting	Expert-rated metrics on empathy, adaptability, communication clarity, therapeutic effectiveness, cultural sensitivity, and trust-building via standardized prompts	Qualitative	N/A
Bird & Lotfi (2023)	7	Dataset of mental health Q&A from verified sources (e.g., NHS, BBR Foundation); chatbot tested with pre-defined questions	Chatbot-based QA system trained on transformer models for depression and anxiety support	Simulated evaluation; hyperparameter tuning experiment	Token prediction accuracy (88.65%), top-5 (96.49%) and top-10 (97.88%) accuracy on chatbot responses	Quantitative	Variants of transformer models (e.g., 2, 4, 8, 16 attention heads; 64–512 neurons); no human comparator

Bird et al. (2024)	8	3508 patient-therapist query-response pairs; patient input + human and AI (Mistral-7B) psychologist responses	Linguistic characteristics of AI vs. human responses in therapeutic dialogues	Synthetic comparative analysis using NLP and computational linguistics	Linguistic feature comparison across 10 dimensions, including: lexical diversity, readability, emotion/sentiment analysis, sentence structure, word usage, punctuation, NER, etc.; analysis used Wilcoxon signed-rank test to examine statistical differences between human and AI responses	Qualitative	N/A
Brocki et al. (2023)	9	Users of the Serena chatbot (no exact number reported); broad demographic expected	AI-mediated person-centered therapy using the Serena dialogue system	System design and simulated deployment	User survey responses (perceived understanding, engagement, helpfulness); internal testing (response coherence, hallucination)	Qualitative	N/A

Chen et al. (2024) D	10	DAIC-WOZ: 189 participants (PHQ-8 label ≥10 as depressed); EATD: 162 participants (SDS≥53)	SEGA: Structural Element Graph for clinical depression detection with LLM-based data augmentation (SEGA++)	Experimental comparison using real-world datasets	Main outcome: Depression classification performance (F1-scores for depressed/control/macro); Evaluation metrics: classification F1	Quantitative	Baselines: ω-GCN, EATD-Fusion, MFM-Att, HCAG, GPT-3.5/4 (zero-/few-shot); traditional models
Chen et al. (2024) E	11	125 Reddit users from the UMD Suicidality Dataset, each with 1–3 posts on r/SuicideWatch subreddit, annotated for suicide risk levels by psychologists	Zero-shot LLM-based evidence extraction using different prompt strategies (baseline, factor-oriented, risk-level & factor-oriented)	Simulated evaluation using shared task benchmark (CLPsych 2024)	BERTScore metrics (recall, precision, weighted-recall) for extracted evidence matching expert-annotated ground truth	Quantitative	Baseline vs. factor-oriented vs. RF-oriented prompts

Danner et al. (2023)	12	Participants from DAIC-WOZ (56 depressed, 133 non-depressed) and Extended-DAIC (66 depressed, 209 non-depressed); simulated interviews with psychology students	Depression detection using BERT, GPT-3.5, and GPT-4 on text-based interview data	Experimental, model comparison	Precision, recall, F1-score; PHQ-8 as ground truth; comparison across real and simulated data	Quantitative	Prior models (e.g., Villatoro-Tello, Senn); GPT-3.5, GPT-4 vs. BERT
De Freitas et al. (2024) – Study 1a & 1b	13	Study 1a: 3,201 conversations from 2,650 Cleverbot users; Study 1b: 17,959 conversations from 10,869 Simsimi users (English users in US, Canada, UK)	Mental health-related conversations with companion AI (e.g., signs of depression, suicidal thoughts, self-harm)	Analysis of real-world chat logs using keyword dictionary and manual coding	Frequency of mental health topics, engagement metrics (duration, number of turns, word length), and chatbot responses (recognition, empathy, helpfulness)	Mixed	None
De Freitas et al. (2024) – Study 2	14	5 commercially available AI companions; 1,080 crisis messages (explicit and vague)	Companion AI's responses to 6 mental health crisis types (depression, suicide, self-injury, harming others, abuse, rape)	Systematic audit using scripted messages; experimental prompt-response testing	Recognition, empathy, provision of mental health resources, overall helpfulness, riskiness of responses	Quantitative	None

De Freitas et al. (2024) – Study 3	15	N = 434 participants from Prolific (Mage = 38, 40% female)	Exposure to helpful vs. unhelpful (risky and non-risky) chatbot responses in mental health crisis contexts	Randomized between-subject online experiment	App usage intention, perceived harm, app rating, liability (reasonable to sue), perceived comprehension; mediation by "potential to cause harm" and "does not comprehend"	Quantitative	Three conditions: helpful, unhelpful–non-risky, unhelpful–risky
Dergaa et al. (2024)	16	3 fictional patients: A (22M, student), B (58F, SLE), C (23F, postpartum)	GPT-3.5 for mental health assessment & recommendations	Simulated interaction study	Evaluated by psychiatrists on accuracy, empathy, appropriateness of advice; focused on limitations in diagnostic reasoning and safety risks in complex cases	Qualitative	N/A
Dongre (2024) Study 1	17	12 undergraduate and 7 graduate students at VT, who spent >10h/week in a university building	Stress due to academic pressure and environmental factors (e.g., thermal comfort)	Focus groups and semi-structured interviews	Thematic analysis identifying "stress" as a major concern; environmental stressors (e.g., thermal discomfort) were emphasized	Qualitative	N/A

Dongre (2024) Study 2	18	8 PhD students (5 men, 3 women, aged 23–37)	EmLLM: A stress-responsive chatbot using wearable data & LLM fine-tuned with mental health dialogues	Pilot study	Outcomes: Stress prediction accuracy (based on CNN model), human-likeness (Godspeed), therapeutic alliance (SRS), perceived usefulness; wearable data collected via Empatica smartwatch	Quantitative	Not reported
D'Souza et al. (2023)	19	Responses of GPT-3.5 3.5 to 100 psychiatric clinical case vignettes; evaluated by 2 expert psychiatrists	AI performance in psychiatric diagnostic and management tasks	Simulated evaluation study	Responses were graded (A–C) by experts based on coverage of 10 themes (e.g., diagnosis, management, reasoning). Grades based on comparison to reference key	Qualitative	N/A

Elyoseph & Levkovich (2023)	20	GPT-3.5 (March 14 version); comparison sample: 379 mental health professionals (21% male, M_age = 36, including psychologists, students, experts, supervisors)	Suicide risk assessment via GPT-3.5 based on text vignettes with manipulated levels of **perceived burdensomeness** and **thwarted belongingness**	Simulated evaluation with vignette-based design and statistical comparison (t-test)	Suicide risk assessment indicators: ① Psychache, ② Suicidal ideation, ③ Suicide attempt risk, ④ Resilience (7-point Likert scale)	Quantitative	Mental health professionals' assessments from Levi-Belz & Gamliel (2016)
Elyoseph & Levkovich (2024)	21	4 LLMs (GPT-3.5, GPT-4, Claude, Bard); 342 nurses, 564 psychiatrists, 424 GPs, 228 clinical psychologists, and 982 general public respondents	Intervention: Use of LLMs to assess prognosis and long-term outcomes of schizophrenia recovery	Case vignette study with ANOVA	Outcome ratings on prognosis (with/without treatment), long-term positive and negative outcomes, and expected discrimination; measured via structured questions rated by LLMs and benchmarked against professional/public data	Quantitative	Human professionals and general public benchmarks

Elyoseph et al. (2024)	22	Four LLMs (GPT-3.5, GPT-4, Claude, Bard); Human data from prior samples: 535 psychiatrists, 211 clinical psychologists, 438 GPs, 328 nurses, 952 general public	Use of LLMs to assess depression prognosis and long-term outcomes based on clinical vignettes	Comparative quantitative analysis using text vignette evaluation; secondary analysis of previously published human data	Prognosis with and without treatment, anticipated long-term positive and negative outcomes, discrimination prediction; scores compared using ANOVA	Quantitative	Human data from prior studies (Caldwell & Jorm), including mental health professionals and general public
Englhardt et al. (2024) Part A	23	90 class-balanced samples (PHQ-4 <1 or >5) from GLOBEM dataset, each includes 28 days × 16 features of passive sensing data from smartphones and wearables	LLM-based classification (e.g., GPT-4, PaLM 2, GPT-3.5) with CoT, Direct Prediction, etc.	Experimental evaluation (zero-shot classification task)	Accuracy of depression and anxiety classification (e.g., 61.11% max); numerical reasoning accuracy (% correct trends, % correct numbers)	Quantitative	Random Forest, Reorder, Reorder-54

Englhardt et al. (2024) Part B	24	8 licensed mental health clinicians (6 PhD, 2 Master's) from diverse U.S. therapy settings, recruited via mailing lists and social media	Clinician perspectives on using LLMs for analyzing passive sensing data collaboratively with patients	Interactive interview + post-survey	Interview responses: perceived usefulness, concerns, envisioned uses (e.g., collaboration, documentation); Post-study survey with Likert responses on trust and utility	Qualitative	N/A
Furukawa et al. (2023)	25	FLATT trial: 164 patients with treatment-resistant depression; HCT trial: 1134 university students with mild depression; total 7182 thought-feeling records	AI-assisted cognitive restructuring using Japanese T5 model in smartphone-based CBT	Secondary analysis of two RCTs with cross-validation machine learning evaluation	Prediction accuracy of AI model; agreement with human expert ratings; reduction in negative emotions via CBT modules	Quantitative	None (no direct intervention comparator; analysis focuses on prediction model accuracy)
Gargari et al. (2024)	26	20 psychiatric clinical cases from the DSM-5 Clinical Cases book; selected by experts for diagnostic diversity	Use of four advanced LLMs (GPT-3.5, GPT-4, Aya, Nemotron) to provide diagnoses and reasoning based on case vignettes	Simulated diagnostic evaluation using text-based prompts	Diagnostic accuracy (correct/partially correct/incorrect), reasoning quality (over-conclusive, supplementary, incomplete)	Quantitative	Other LLMs (models compared with one another); no human clinician control group

Giorgi et al. (2024)	27	150 real-world Reddit questions (25 each from r/stopdrinking, r/leaves, r/OpiatesRecovery); evaluated by clinicians	Effectiveness, factuality, and safety of AI-generated responses to real-world SUD-related questions	Qualitative content analysis and clinical fact-checking	Fact-checking dangerous advice (e.g., home detox, microdosing); inconsistency analysis via rephrasing and reprompting; quotes analyzed thematically	Qualitative	N/A
Giorgi et al. (2024) Part A	28	7 clinicians evaluated 150 AI responses to 75 Reddit questions on substance use and recovery	AI-generated answers from GPT-4 and LLaMA-2 to real-world SUD-related questions	Simulated evaluation with clinician ratings	Clinician ratings on adequacy (1–3), appropriateness (1–5), and overall quality (1–5); inter-rater agreement; % below midpoint scores; significant differences across conditions	Quantitative	GPT-4 vs LLaMA-2

Hadar-Shoval et al. (2023)	29	Simulated responses by GPT-3.5 3.5 to 20 LEAS scenarios framed as if experienced by individuals with BPD and SPD (no human participants involved)	The phenomenon of interest is GPT-3.5's ability to simulate **mentalizing-like emotional awareness** responses tailored to BPD and SPD personality structures	Simulated evaluation using AI model	Evaluation based on LEAS (Levels of Emotional Awareness Scale) scores, number of emotions, and emotional intensity in GPT-3.5 responses under BPD and SPD conditions	Qualitative	N/A
Hayati et al. (2022)	30	N=53 Malaysian adults speaking KL, Pahang, or Terengganu dialects; 11 with clinically confirmed depression	GPT-3-based depression detection using few-shot learning on Malay dialectal speech	Experimental classification (machine learning deployment)	Depression detection performance measured by macro F1 score and accuracy across different dialect groups and example sizes	Quantitative	None (no comparison to other models in this study)
Hedderich et al. (2024)	31	13 middle school teachers from the US, Canada, and Southeast Asia, with varied backgrounds in digital citizenship, health, and technology.	Teachers' needs and practices in designing LLM-powered chatbots to teach adolescents cyberbullying upstanding behavior.	Think-aloud protocol, contextual inquiry, and semi-structured interviews using a design probe (Co-Pilot).	Teachers' experiences, needs, and feedback when building/testing chatbots; observations and interview transcripts analyzed using affinity	Qualitative	N/A

					diagramming to derive themes and patterns.		
Heinz et al. (2023)	32	N = 1710 GPT-3 diagnostic outputs generated in response to 59 contrived clinical vignettes programmatically varied by race, sex, and age	Use of GPT-3 (a large language model) to assess diagnostic accuracy and bias in mental health interpretation of clinical vignettes	Simulated evaluation using contrived vignettes	Balanced Accuracy (BAC), sensitivity, specificity, PPV, NPV for diagnosis; Demographic bias assessed via odds ratios using generalized linear mixed-effects models across race, sex, and age	Quantitative	None (Not reported)
Herencia (2024)	33	Conversational data from mental health forums (quantity not specified)	Fine-tuned LLaMA model for mental health counseling via LoRA	Simulated evaluation	BERTScore, METEOR Score, response inference time	Quantitative	Original non-fine-tuned LLaMA

Heston (2023)	34	25 GPT-3.5-based conversational agents from FlowGPT.com repository, designed for mental health counseling	Evaluation of safety and crisis response behavior of LLM-based mental health chatbots using simulated depression and suicidality prompts	Simulated evaluation (observational, cross-sectional)	Initial referral point to human support; shutdown point; whether crisis resources (e.g., suicide hotlines) were offered; whether agent resumed conversation	Quantitative	None
Hodson & Williamson (2024)	35	Two CBT therapists (NHS, UK); each generated 10 vignettes (total 20); LLMs were tested on these tasks	Use of LLMs (GPT-4, Bard) to perform CBT tasks: Catch it, Check it, Change it	Simulated evaluation with expert rating	Accuracy in identifying cognitive biases and reframing thoughts: GPT-4 scored 44/60; Bard scored 42/60; therapist agreement (Cohen's κ = 0.44) noted as moderate	Quantitative	None (no traditional CBT or human-only comparator)
Hsieh et al. (2023)	36	1 doctoral counseling student, 6 doctoral-level counseling psychologists, GPT-3.5 output, 1 client case	Use of GPT-3.5 for training case conceptualization skills in counseling	Single-blind qualitative evaluation	Evaluated by experts using 7-point Likert scale on accuracy, completeness, feasibility, and consistency; qualitative feedback also collected	Qualitative	N/A

Hu et al. (2024)	37	Data from 3 multimodal datasets: • MODMA: patients with MDD and controls• PME4: 11 acting students (5F, 6M)• LUMED-2: emotion-labeled data with facial expression and EEG	MultiEEG-GPT method using LLMs (GPT-4o) with EEG + audio or EEG + facial expressions under zero-shot and 1-shot prompting	Single modality settings (EEG only, audio only, or facial expression only), and zero-shot vs. few-shot prompting	Prediction accuracy in multi-class classification of mental health status: depression (MODMA) and emotions (PME4, LUMED-2); evaluated by accuracy (%) across 5 trials per condition	Quantitative	Simulated evaluation (zero-shot & few-shot classification on existing datasets)
Hu et al. (2024) - Qualitative	38	10 Chinese children (aged 8–12) and their parents	Children's needs and preferences in mental resilience education using AI conversational agents	Qualitative interview + field observation	Semi-structured interviews and observations analyzed via coding and clustering to extract design challenges	Qualitative	N/A
Hu et al. (2024) - Quantitative	39	48 children (aged approx. 11–18)	LLM-powered conversational agent for resilience cultivation, emotional support, and psychological measurement	3 experiments: (1) pre-post intervention trial, (2) crossover comparison with traditional scale, (3) system	Traditional scale (for Exp 2); None (for Exp 1 & 3)	Qualitative	N/A

				usability assessment			
Hur et al. (2024)	40	N = 467 (Study 1: 179, Study 2: 288); general adult population recruited via Prolific; mean age ≈ 43	Intervention: Brief open-ended text responses analyzed for language sentiment using human raters, GPT-3.5 (GPT-3.5, 4.0), and LIWC	Longitudinal study (baseline & 3-week follow-up); quantitative analysis	Outcome: Change in depressive symptoms (PHQ-9) at 3-week follow-up; predictors: language sentiment ratings (human/AI/LIWC)	Quantitative	None (no control group; compared models with vs. without sentiment scores)
Hwang et al. (2024)	41	One fictional case (Ms. Z, a female artist with panic attacks and depression) from psychoanalytic literature; evaluated by five psychiatrists from varied settings	Use of GPT-3.5 to generate psychodynamic formulations of a patient	Qualitative evaluation using expert judgment; exploratory design with prompt variation	Appropriateness of GPT-3.5 outputs (rated by psychiatrists); interrater agreement measured; dimensions include theoretical depth, model consistency, and	Qualitative	N/A

					alignment with clinical formulations		
James et al. (2024) - Qualitative	42	13 participants (9 case managers + 4 health professionals)	Perceptions of GOALS system and workflow	Thematic analysis from post-study survey & researcher notes	Themes: (1) Language accessibility; (2) Workflow usability; Subthemes include speed, professionalization, resistance to AI, patient preference	Qualitative	N/A
James et al. (2024) - Quantitative	43	9 case managers from FACT teams in the Netherlands	LLM-powered goal generation system (GOALS) for measurable treatment goals in patients with SMI	Within-subject experimental design	GOALS rubric scores (SDT & GST elements); statistical analysis (t-test, Cohen's D) to compare LLM vs human-generated goal quality	Quantitative	Case manager-created goals

Kim et al. (2024a)	44	N = 19 OCD vignettes and 7 control vignettes; the LLMs tested include GPT-4, Gemini Pro, and Llama 3.	LLM-based OCD diagnosis via vignettes	Case-control design (vignette-based)	Diagnostic accuracy of LLMs vs. human professionals; measured by correct primary diagnosis identification	Quantitative	Mental health providers, medical doctors, clergy, doctoral students in psychology
Kim et al. (2024b) - Qualitative	45	6 Mental Health Professionals (4 clinical psychologists, 2 psychiatrists)	Use of LLMs in clinical mental health support and journaling	Focus group interview	Thematic analysis of clinicians' views: challenges in patient communication, potential of LLMs in journaling	Qualitative	N/A
Kim et al. (2024b) - Quantitative	46	28 psychiatric outpatients (age 12–28, diagnosed with MDD)	LLM-based journaling via MindfulDiary	Field deployment study	Journaling adherence, emotional self-expression, PHQ-9 trends, patient-clinician communication	Quantitative	None

Kumar et al. (2023)	47	189 participants from Amazon Mechanical Turk; adults aged 18+, majority with prior mindfulness experience (93.12%), majority employed full-time and with Bachelor's degree or higher	Three interventions: (1) Informational Chatbot (GPT-3-based), (2) Tutorial Video on mindful breathing, and (3) Reflection Chatbot (GPT-3-based). All evaluated in 2x2x2 randomized factorial design.	Randomized Controlled Trial (2x2x2 between-subject factorial experiment)	(1) Self-reported intent to practice mindfulness again (1–7 scale); (2) Overall experience rating of the intervention (1–7 scale)	Quantitative	Text-only description of mindfulness (baseline, no chatbot or video)
Lee et al. (2024)	48	460 telemental health patients (140 SI at intake, 120 SI post-intake, 200 no SI); adults using Brightside platform; de-identified intake data used	GPT-4-based SI with plan prediction from intake text	Comparative study (experimental benchmarking)	Sensitivity, precision, specificity, accuracy of SI with plan prediction at intake and post-intake by GPT-4 vs. clinicians	Quantitative	Senior clinicians' predictions (n=6)

Levkovich & Elyoseph (2023)	49	GPT-3.5 (May 24) and GPT-4 (May 24); comparison with 379 mental health professionals (Israel; mean age 36; majority practicing psychologists)	AI-based suicide risk assessment using GPT-3.5 and GPT-4 based on vignettes with manipulated levels of perceived burdensomeness and thwarted belongingness	Vignette-based simulated evaluation; repeated measures with both models	Z-score and mean comparisons across 4 conditions on 4 outcomes: suicidal ideation, suicide attempt risk, psychache, and resilience	Quantitative	Mental health professionals' average ratings; GPT-3.5 (March 14 version)
Levkovich & Elyoseph (2023)	50	- GPT-3.5 3.5 & 4 evaluated using 8 vignettes (with variations by gender, SES, depression severity) - Compared to norms from 1249 primary care physicians (72.9% female)	- GPT-3.5-generated treatment suggestions for mild/severe depression (psychotherapy, medication, or both)	Simulated evaluation using vignettes	- Frequency of therapy types suggested (psychotherapy only, pharmacology only, combination) - Differences in recommendations by depression severity, gender, SES - Consistency with clinical guidelines - Comparison via chi-square statistical analysis	Quantitative	Primary care physicians' responses (n = 1249)

Li et al. (2024)	51	Publicly available social media datasets: Depression_Reddit and IRF; no human subjects directly involved	Intervention: MAIMS (Mental Analysis by Incorporating Mental Scales), a two-step LLM-based method	Simulated evaluation	Weighted F1-score, accuracy on mental health classification tasks; interpretability of explanations; ablation study results	Quantitative	Compared against baseline models: BERT, RoBERTa, GPT-3.5, GPT3.5, MentaLLaMA, etc.
Liu et al. (2024)-Quantitative	52	Two datasets: (1) 9,257 HGC texts from One Psychology platform; (2) 9,257 AIGC texts by GPT-3.5	Use of GPT-3.5-generated content for depression intervention	Quantitative study using machine learning (BERT, Roberta, TextCNN, LSTM)	Accuracy (up to 93.76%), F1-score, linguistic comparison, SHAP-based interpretability, intervention utility (e.g., supportiveness of responses)	Quantitative	Human-generated content (HGC) responses

Ma et al. (2023)	53	120 Reddit posts, 2917 comments from 462 unique users on r/Replika subreddit	User experiences with LLM-based conversational agents (Replika) for mental well-being support	Qualitative content analysis of Reddit posts	Thematic coding of perceived benefits (e.g., on-demand and non-judgmental support, increased confidence, self-discovery) and challenges (e.g., harmful content, memory failure, inconsistent styles, overreliance, social stigma). User narratives were analyzed using open coding and a revised codebook.	Qualitative	N/A
Ma et al. (2024)	54	31 participants (18 LGBTQ+, 13 non-LGBTQ+); mostly U.S.-based adults aged 22–36, frequent chatbot users	Use of LLM-based chatbots for emotional and mental health support among LGBTQ+ users	Qualitative interviews (semi-structured)	Thematic analysis of interview transcripts focusing on accessibility, emotional support, identity exploration, safety, and perceived limitations of LLMs	Qualitative	N/A

Maurya et al. (2024)	55	GPT-3.5 responses (N=21 prompts); prompts created by researchers with mental health counselling experience	Use of GPT-3.5 as a psychoeducational tool for addressing mental health concerns	Qualitative content analysis (QCA); deductive coding framework	6 dimensions of assessment: accuracy, clarity, relevance, empathy, engagement, and ethical considerations. Evaluation via a 5-point scale and thematic interpretation of responses.	Qualitative	N/A
Mazumdar et al. (2023)	56	Reddit users; dataset contains social media posts labeled for mental health risk factors.	GPTFX: Mental health detection using GPT embeddings and GPT-3 explanation.	Experimental evaluation using public dataset	Classification accuracy (up to 87.34%) and explanation quality (ROUGE-1 up to 0.7596, BLEU-1, EM, Cosine similarity). Evaluated on two labels: *Thwarted Belongingness* and *Perceived Burdensomeness*.	Quantitative	Compared against traditional models (LIME, SHAP) and PLMs (MentalBERT, PsychBERT, ClinicBERT, BERT)

Meyer & Elsweiler (2024)	57	164 German-speaking participants (aged 19–72, M=32.28, 41.4% female); recruited via Prolific	GPT-4-based chatbot providing motivational interviewing (MI)-style behavior change support (vs. non-MI GPT-4 chatbot)	Between-subject field experiment (random assignment to 2 chatbot conditions)	Change in readiness to change (pre-post), perceived empathy, therapeutic alliance (WAI-SR), MI adherence (CEMI), user engagement (UES-SF), perceived communication competence	Quantitative	Non-MI GPT-4 chatbot
Najarro et al. (2023)	58	No empirical sample (conceptual system design); future work mentions surveys and user studies	AI-mediated 24/7 proactive mental health counseling via WMGPT (GPT-3.5-based system)	Conceptual system design; prototype architecture	No real-world evaluation yet; planned future outcomes: stress/depression detection accuracy, user self-reflection, help-seeking behavior	Qualitative	N/A
Ni et al. (2024a) – Qualitative	59	6 participants (3 females, 3 males; aged 16–35) in unstructured discussion for needs analysis (diverse age and regional backgrounds)	User needs and preferences in psychoecological data collection via chatbot	Qualitative interview; modified affinity diagram method	User-reported needs on deployment, usability, and privacy; categorized and visualized with affinity diagram	Qualitative	N/A

Ni et al. (2024b) – Quantitative	60	12 participants (7 males, 5 females; mean age = 23.1) for usability testing; 317 adolescents (mean age = 12.3) in ILD deployment phase	Chatbot-based psychological scale administration and QA (Beatrice)	System usability test (SUS); longitudinal deployment in natural settings	SUS score (mean = 85.4); 4675 valid responses collected; feedback from teachers; tool improvements tracked through feature requests	Quantitative	Not reported
Ohse et al. (2024)	61	N = 82; general German population; age range 19–75 (M = 31.64, SD = 11.81)	Depression detection via LLMs (GPT-4, GPT-3.5, BERT, Llama2)	Cross-sectional experimental	Main outcomes: F1 score, precision, recall, PCC, MSE, CV; Depression classification using PHQ-8 as ground truth from clinical interview transcriptions.	Quantitative	None (no human or traditional method comparator was tested); comparison is between models
Perlis et al. (2024)	62	50 clinical vignettes for individuals with bipolar I or II depression; 3 expert psychiatrists; 27 community clinicians	Intervention: Augmented LLM (GPT-4-turbo) using evidence-based treatment guidelines for next-step pharmacotherapy selection	Simulated evaluation study	Primary outcome: Agreement with expert-defined optimal treatment (Cohen's kappa, top 1/3/5 match rates); secondary: selection of poor treatments	Quantitative	Unaugmented LLM (GPT-4-turbo without guidelines), Community clinicians (psychiatrists and other prescribers)

Pugh et al. (2024)	63	51 participants: 26 patients with schizophrenia or schizoaffective disorder; 25 healthy controls	Intervention: Use of LLMs (GPT-3.5, GPT-4, Llama 3) to predict thought disorder dimensions (coherence, content, tangentiality)	Comparative computational study with re-analysis of prior dataset + experiments with 2 & 25 iterations	Accuracy: Correlation between LLM-generated and human expert ratings; Consistency: Variability across trials and model parameters; Ensemble effectiveness in improving consistency and accuracy	Quantitative	Supervised machine learning baseline; No-treatment control group (human ratings as ground truth)
Radwan et al. (2024)	64	>10,000 labeled social media posts (Reddit); user demographics: 56% women, 44% men; age 18–55+; mainly North America (85%) and Europe (11%)	GPT-3 embeddings + ML classifiers (SVM, RF, etc.) to detect stress-related posts	Quantitative study; secondary data analysis using supervised machine learning and NLP	Accuracy, precision, recall, F1-score; SVM achieved 83% accuracy in classifying stress-related posts	Quantitative	Compared with BERT + LSTM, Metapath2Vec + Logistic Regression, mBERT + CNN-BiLSTM
Saleem & Kim (2024)	65	DREADDIT dataset: 3,000 Reddit posts (2,838 for training, 715 for testing), labeled as “stressed” or “not stressed”	Intent-aware 1-shot data augmentation using GPT-3.5 (same and opposite intent prompts)	Experimental comparison (NLP model evaluation)	F1-score, precision, recall, accuracy of stress classification using RoBERTa	Quantitative	Back Translation, EDA, AEDA, RoBERTa without augmentation

Sharma et al. (2024) - Qualitative	66	Subset of participants who provided qualitative feedback (open-ended)	Human–LLM interaction in supporting emotional and cognitive challenges	Open-ended qualitative survey post-intervention	Thematic feedback on usability, emotional comfort, perceived support, interactivity, and limitations (e.g., simplicity)	Qualitative	N/A
Sharma et al. (2024) - Quantitative	67	15,531 participants (13+ yrs), users of a public mental health website (MHA), diverse in age/gender/education	AI-assisted cognitive restructuring via LLM (GPT-3 based system)	RCT in field setting	Emotion intensity reduction, reframe helpfulness, relatability, memorability, and skill learnability (quantified via scales)	Quantitative	Design ablations (e.g., without psychoeducation, without interactivity)
Shin et al. (2024)	68	91 participants (mostly women aged 20–39), 428 diary entries via emotional diary-writing app	Depression detection via diary text analysis using LLMs (GPT-3.5, GPT-4)	Instrument validation study	Depression risk prediction accuracy, recall, specificity, F1 score (e.g., GPT-3.5 accuracy = 0.902, recall = 0.643–0.929)	Quantitative	None

Shinan-Altman et al. (2024)	69	160 evaluations across 8 vignettes (2×2×2 design: gender, history of depression, access to weapons); each vignette evaluated 10 times by GPT-3.5 and 10 times by GPT-4	Suicide risk assessment by GPT-3.5 vs. GPT-4 based on vignettes involving depression and weapon access	Simulated evaluation using vignettes with multivariate 3-way ANOVA	Likelihood of suicidal thoughts, suicide attempts, serious suicide attempts, and suicide-related mortality, rated on an 8-point Likert scale by GPT-3.5 models	Quantitative	GPT-3.5 vs. GPT-4 (internal comparator)
Singh et al. (2024) - Qualitative	70	None reported (no empirical participants; system architecture and demo design only)	AI-mediated mental health support via MindGuide chatbot based on LangChain and GPT-4	System architecture and demonstration study	Demonstration of chatbot features, including early detection capabilities, context-aware reasoning, memory, and user interaction via Streamlit; qualitative description of interaction design	Qualitative	N/A

Singh et al. (2024) - Quantitative	71	Reddit users from r/SuicideWatch subreddit; annotated posts (UMD Suicidality Dataset); no specific N reported	Use of LLMs (Mixtral7bx8, Tulu-2-DPO-70B) for extracting and summarizing suicidal ideation evidence using prompting strategies (Zero-shot, Few-shot, CoT)	Simulated evaluation using CLPsych 2024 shared task benchmark	F1-score for evidence extraction; consistency score for summarization; impact of meta-information	Quantitative	BERT-based baseline and other LLM-based teams (e.g., sophiaADS, UZH_CLyp)
Smith et al. (2023)	72	No human participants; interactions were between researchers and GPT-3.5.	Use of GPT-3.5 to support educational methods in social psychiatry.	Exploratory simulation study	Evaluation based on GPT-3.5's responses to prompts (e.g., case vignette creation), judged by researchers based on relevance, feasibility, and pedagogical utility.	Qualitative	N/A
Soun & Nair (2024)	73	Reddit users from three datasets (CLPsych: 11,129 users; Dreaddit: 3,553 labeled posts; Suicide Risk Dataset: 500 users). Age and	Intervention: Using GPT-3.5 turbo to classify suicide risk severity, suicide ideation, and stress from Reddit posts	Simulated evaluation	Accuracy and F1 score on multi-class and binary classification tasks across demographic groups	Quantitative	Traditional ML models (MLP, LSTM, T-LSTM, FAST, GPols, BERT Base)

		gender groups analyzed.					
Stern et al. (2024) - Qualitative	74	5 psychology PhD students (user study), each reviewed multiple Reddit posts	Human expert perception of LLM-generated suicide risk explanations	Qualitative user study with multi-round task design	Human-labeled risk classifications and qualitative comparison with model output	Qualitative	N/A
Stern et al. (2024) - Quantitative	75	484 Reddit posts from UMD dataset (crowdsourced)	Use of LLMs (e.g., LLaMA, WizardLM, SpeechlessLM) for suicide risk classification	Quantitative benchmark with BLEU/ROUGE and classification metrics	F1, Precision, Recall; BLEU/ROUGE scores for explanation quality	Quantitative	Compared to MentalBERT baseline

Tao et al. (2023)	76	139 patients (64 with depression, 75 with anxiety) from Peking University Sixth Hospital, China; age ~32–38 years	Use of a virtual interaction framework with GPT-3.5 for classifying anxiety vs. depression based on real-life Q&A dialogues and speech data	Experimental case study using real Q&A data and multimodal feature prompting (e.g., speech rate, rhythm)	Classification accuracy of GPT-3.5 in distinguishing anxiety and depression using various text and speech prompts (e.g., 79.14% with rhythm info)	Quantitative	None (no traditional method or other AI model used as baseline)
Uluslu et al. (2024)	77	125 Reddit users with at least one post in r/SuicideWatch, annotated with risk levels (No, Low, Moderate, Severe)	Suicide risk detection using Retrieval-Augmented Generation (RAG) with emotion regression in LLMs	Shared Task Evaluation using benchmark dataset (CLPsych 2024), zero-shot prompting with Mistral-7B-Instruct model	Task A: Snippet extraction evaluated by precision, recall, weighted recall using BERTScoreTask B: Summary generation evaluated by mean consistency and max contradiction against expert annotation	Quantitative	Ablation studies (No Emotion Regression, No Emotion RAG); baseline models from other teams

Vakayil et al. (2024)	78	Victims of sexual harassment in India (target user group); no actual user sample tested	Use of LLM-powered RAG-based chatbot (Llama-2) to provide empathetic and legal support	Simulated evaluation (technical design + scenario-based testing)	Evaluation through chatbot's response quality: empathy, contextual appropriateness, accuracy (>95%), supportiveness, correctness in identifying harassment types	Qualitative	N/A
Vowels et al. (2024)	79	20 UK participants (8 men, 12 women, aged 25–62, average age = 41.8), in relationships (avg. 11.2 years)	AI-mediated single-session relationship support via GPT-3.5 (GPT-4)	Qualitative interview study	Thematic analysis of user experiences; content analysis of GPT-3.5's performance (e.g., empathy, flow, questioning); user–researcher agreement on 21 coded metrics	Qualitative	N/A
Wang et al. (2023)	80	50 mental health interns participated in the model evaluation; other parts used datasets only	Knowledge-enhanced pre-training of LLMs for depression diagnosis and treatment	Technical framework and simulation-based evaluation	Evaluation based on **safety**, **usability**, and **fluency** using 1–10 ratings from 50 interns after model interaction	Qualitative	N/A

Wu et al. (2024)	81	219 participants from E-DAIC dataset (49 PTSD-positive, 114 PTSD-negative); gender-balanced; age ≈ 50 ± 21	Data augmentation using LLMs (CALLM framework with Response-Reason prompts and T-A-A design)	Experimental (ML-based simulated evaluation using real and synthetic data)	PTSD classification performance: balanced accuracy = 0.77, F1 = 0.70, AUC = 0.78; model = DistilBERT fine-tuned on synthetic and real data	Quantitative	Compared with E-DAIC baseline, SMOTE, Back Translation, Text Replacement, prior studies (Galatzer-Levy et al., Flores et al.)
Wu et al. (2024) Part B	82	25 participants (13 female, 12 male; mean age ≈ 22; students and young professionals with high smartphone usage and willingness to reduce it)	MindShift: LLM-powered intervention generating personalized persuasive content based on user context and mental states	5-week within-subject field experiment	intervention acceptance rate, app usage frequency and duration, Smartphone Addiction Scale (SAS), self-efficacy scale, user feedback	Quantitative	MindShift-Simple; Baseline reminder
Wu et al. (2024) Part A	83	12 participants in Wizard-of-Oz study (6M/6F, 18–28); 10 participants in interviews (5M/5F, 18–29)	Mental states behind habitual/problematic smartphone use (e.g., boredom, stress, inertia)	Wizard-of-Oz field study and semi-structured interviews	Thematic coding of mental states; participant feedback on message acceptance; qualitative insights on user reactions to persuasive strategies	Qualitative	N/A

Xu et al. (2024)	84	Online posts from Reddit, Twitter, and SMS-style datasets; data annotated by experts. Example datasets include Dreaddit (N≈3600), DepSeverity (N≈3550), SDCNL (N≈1900), CSSRS (N=500), etc.	Instruction-finetuning of LLMs (e.g., Alpaca, FLAN-T5) for mental health prediction (stress, depression, suicide ideation/risk)	Large-scale benchmark experiment (quantitative, multi-model comparison)	Balanced accuracy across six mental health prediction tasks (e.g., stress, depression, suicide ideation/risk). Performance compared across prompting methods (zero-shot, few-shot) and fine-tuned models (Mental-Alpaca, Mental-FLAN-T5).	Quantitative	Baselines include GPT-3.5, GPT-4, BERT, Mental-RoBERTa
Yahagi et al. (2024)	85	100 adult patients undergoing surgery under general anesthesia (final N=85; Control=44, Intervention=41); mean age ~54–61	GPT-3.5-based preoperative education (AI chatbot intervention)	Randomized Controlled Trial (single-blind)	Primary: Preoperative anxiety (STAI); Secondary: Satisfaction, understanding, perceived relevance of information	Quantitative	Standard anesthesia nurse education

Yang et al. (2024)	86	105K samples from 10 datasets (e.g., Reddit, Twitter, SMS); covering 8 mental health analysis tasks (e.g., depression, stress, loneliness); data labeled with binary or multi-class annotations, often via human or weak supervision.	Intervention: Fine-tuning open-source LLaMA2 models (7B/13B) using IMHI dataset to build MentaLLaMA models for interpretable mental health analysis on social media.	Experimental benchmarking with finetuning + evaluation on standardized benchmark (IMHI test set); includes generalization tests to unseen tasks.	Correctness (weighted F1 scores), explanation quality (BART-score), human evaluation (consistency, reliability, professionality, overall quality); both automatic and human evaluations used. Explanations generated by GPT-3.5 and evaluated; final model (MentaLLaMA) compared with multiple baselines.	Quantitative	MentalBERT, MentalRoBERTa, BART, T5, GPT-3.5, GPT-4
Yu & McGuinness (2024)	87	10 users with self-reported mental health issues; 10 mental health professionals (researchers and carers)	Fine-tuned DialoGPT model + GPT-3.53.5 prompt injection for mental health support	Experimental study (systematic evaluation + human evaluation)	Perplexity, BLEU scores; user satisfaction (e.g., usefulness, human-likeness, supportiveness)	Quantitative	GPT-3.5 only; Fine-tuned DialoGPT only

Zhang et al. (2024a)	88	10 university students (from original N ≈ 150); age group: young adults; location: Australia	Predicting weekly affective states using smartphone sensor data and LLM-based methods	Quantitative exploratory study; repeated train-test splits; zero-shot & few-shot LLM tasks	Mean Absolute Error (MAE), Relative Error (ε) across 10 I-PANAS-SF items (e.g., Active, Afraid); model prediction accuracy	Quantitative	Zero-shot vs Few-shot
Zhang et al. (2024b)	89	25,051 Reddit posts with emojis (2,329 suicide-related, 22,722 control group)	Emoji-aware suicidal ideation detection and emoji prediction using PLMs and LLMs	Quantitative benchmark evaluation	SID task: Recall and weighted F1 for suicide/average; EP task: micro, macro, and weighted F1 for emoji prediction	Quantitative	Multiple: – Emoji vs no emoji– PLMs (BERT, MentalBERT) vs LLMs (Falcon, LLaMA, GPT-3.5)
Zhou et al. (2023)	90	1462 female firearm suicide decedents (NVDRS, 2014–2018); narrative reports in English	Use of LLMs (FLAN-UL2) to identify rare pre-suicide circumstances	Quantitative evaluation using NLP classification tasks	F1-scores for identifying 9 infrequent circumstances (e.g., sexual violence, sleep problems, bullying)	Quantitative	Conventional supervised machine learning (Support Vector Machine)

Zhu et al. (2024)	91	Reddit users from the r/SuicideWatch subreddit; posts annotated as Low/Moderate/High risk level	Using healthcare-oriented LLMs (XinHai) with prompt engineering to highlight suicide-related evidence in posts	Simulated evaluation based on CLPsych 2024 Shared Task	Phrase-level and sentence-level evidence highlighting performance using BERTScore (Recall, Precision, Harmonic Mean), and summary consistency/contradiction via NLI model	Qualitative	N/A

Reference

GLM for Mental Health Diagnosis and Assessment

Alhamed, F., Ive, J., & Specia, L. (2024). Using LLMs to extract evidence from pre-annotated social media data. Proceedings of the 9th Workshop on Computational Linguistics and Clinical Psychology.

Bauer, B., Norel, R., Leow, A., Abi Rached, Z., Wen, B., & Cecchi, G. (2024). Using large language models to understand suicidality in a social media_based taxonomy of mental health disorders: Linguistic analysis of Reddit posts. JMIR Mental Health, 11, Article e57234. https://doi.org/10.2196/57234.

Chen, Z., Deng, J., Zhou, J., Wu, J., Qian, T., & Huang, M. (2024b). Depression detection in clinical interviews with LLM-empowered structural element graph. Proceedings of the 2024 Conference of the North American Chapter of the Association for Computational Linguistics: Human Language Technologies.

Chen, J., Nguyen, V., Dai, X., Molla, D., Paris, C., & Karimi, S. (2024a). Exploring instructive prompts for large language models in the extraction of evidence for supporting assigned suicidal risk levels. Proceedings of the 9th Workshop on Computational Linguistics and Clinical Psychology.

Danner, M., Hadzic, B., Gerhardt, S., Ludwig, S., Uslu, I., Shao, P., ... & Ratsch, M. (2023). Advancing mental health diagnostics: GPT-based method for depression detection. 2023 62nd Annual Conference of the Society of Instrument and Control Engineers (SICE).

D’Souza, R. F., Amanullah, S., Mathew, M., & Surapaneni, K. M. (2023). Appraising the performance of GPT-3.5 in psychiatry using 100 clinical case vignettes. Asian Journal of Psychiatry, 89, 103770.

Elyoseph, Z., & Levkovich, I. (2023). Beyond human expertise: The promise and limitations of GPT-3.5 in suicide risk assessment. Frontiers in Psychiatry, 14, 1213141. https://doi.org/10.3389/fpsyt.2023.1213141

Englhardt, Z., Ma, C., Morris, M. E., Chang, C. C., Xu, X. O., Qin, L., ... & Iyer, V. (2024). From classification to clinical insights: Towards analyzing and reasoning about mobile and behavioral health data with large language models. Proceedings of the ACM on Interactive, Mobile, Wearable and Ubiquitous Technologies, 8(2), 1-25.

Gargari, O. K., Fatehi, F., Mohammadi, I., Firouzabadi, S. R., Shafiee, A., & Habibi, G. (2024). Diagnostic accuracy of large language models in psychiatry. Asian Journal of Psychiatry, 100. https://doi.org/10.1016/j.ajp.2024.104168

Hayati, M. F. M., Ali, M. A. M., & Rosli, A. N. M. (2022). Depression Detection on Malay Dialects Using GPT-3. 7th IEEE-EMBS Conference on Biomedical Engineering and Sciences.

Heinz, M. V., Bhattacharya, S., Trudeau, B., Quist, R., Song, S. H., Lee, C. M., & Jacobson, N. C. (2023). Testing domain knowledge and risk of bias of a large-scale general artificial intelligence model in mental health. Digital Health, 9, 20552076231170499. https://doi.org/10.1177/20552076231170499

Hu, Y., Zhang, S., Dang, T., Jia, H., Salim, F. D., Hu, W., & Quigley, A. J. (2024). Exploring large-scale language models to evaluate EEG-based multimodal data for mental health. 2024 ACM International Joint Conference on Pervasive and Ubiquitous Computing. https://doi.org/10.1145/3675094.3678494

Hur, J. K., Heffner, J., Feng, G. W., Joormann, J., & Rutledge, R. B. (2024). Language sentiment predicts changes in depressive symptoms. Proceedings of the National Academy of Sciences, 121(39), e2321321121.

Kim, J., Leonte, K. G., Chen, M. L., Torous, J. B., Linos, E., Pinto, A., & Rodriguez, C. I. (2024a). Large language models outperform mental and medical health care professionals in identifying obsessive-compulsive disorder. NPJ Digital Medicine, 7(1), 193.

Lee, C., Mohebbi, M., O'Callaghan, E., & Winsberg, M. (2024). Large language models versus expert clinicians in crisis prediction among telemental health patients: Comparative study. JMIR Mental Health, 11(1), e58129.

Levkovich, I., & Elyoseph, Z. (2023). Identifying depression and its determinants upon initiating treatment: GPT-3.5 versus primary care physicians. Family Medicine and Community Health, 11(4).

Li, W., Zhu, Y., Lin, X., Li, M., Jiang, Z., & Zeng, Z. (2024). Zero-shot explainable mental health analysis on social media by incorporating mental scales. ACM Web Conference 2024. https://doi.org/10.1145/3589335.3651584

Mazumdar, H., Chakraborty, C., Sathvik, M. S. V. P. J., & Panigrahi, P. K. (2023). GPTFX: A novel GPT-3 based framework for mental health detection and explanations. IEEE Journal of Biomedical and Health Informatics.

Ni, Y., Chen, Y., Ding, R., & Ni, S. (2023, July). Beatrice: A Chatbot for Collecting Psychoecological Data and Providing QA Capabilities. Proceedings of the 16th International Conference on PErvasive Technologies Related to Assistive Environments.

Ohse, J., Hadžić, B., Mohammed, P., Peperkorn, N., Danner, M., Yorita, A., ... & Shiban, Y. (2024). Zero-Shot strike: Testing the generalisation capabilities of out-of-the-box LLM models for depression detection. Computer Speech & Language, 88, 101663.

Pugh, S. L., Chandler, C., Cohen, A. S., Diaz-Asper, C., Elvev_g, B., & Foltz, P. W. (2024). Assessing dimensions of thought disorder with large language models: The tradeoff of accuracy and consistency. Psychiatry Research, 341. https://doi.org/10.1016/j.psychres.2024.116119

Radwan, A., Amarneh, M., Alawneh, H., Ashqar, H. I., AlSobeh, A., & Magableh, A. A. A. R. (2024). Predictive analytics in mental health leveraging llm embeddings and machine learning models for social media analysis. International Journal of Web Services Research (IJWSR), 21(1), 1-22.

Saleem, M., & Kim, J. (2024). Intent aware data augmentation by leveraging GAI for stress detection in social media texts. PeerJ Computer Science, 10, e2156.

Shin, D., Kim, H., Lee, S., Cho, Y., & Jung, W. (2024). Using large language models to detect depression from user-generated diary text data as a novel approach in digital mental health screening: Instrument validation study. Journal of Medical Internet Research, 26, e54617.

Shinan-Altman, S., Elyoseph, Z., & Levkovich, I. (2024). The impact of history of depression and access to weapons on suicide risk assessment: a comparison of GPT-3.5 and GPT-4. PeerJ, 12, e17468.

Singh, L. G., Mao, J., Mutalik, R., & Middleton, S. (2024). Extracting and Summarizing Evidence of Suicidal Ideation in Social Media Contents Using Large Language Models. Proceedings of the 9th Workshop on Computational Linguistics and Clinical Psychology.

Soun, R. S., & Nair, A. (2024). GPT-3.5 for mental health applications: A study on biases. Proceedings of the Third International Conference on AI-ML Systems, Bangalore. https://doi.org/10.1145/3639856.3639894

Stern, W., Goh, S. J., Nur, N., Aragon, P. J., Mercer, T., Bhattacharyya, S., ... & Van Minh Nguyen. (2024). Natural language explanations for suicide risk classification using large language models. AAAI (pp. 74-83).

Tao, Y., Yang, M., Shen, H., Yang, Z., Weng, Z., & Hu, B. (2023). Classifying anxiety and depression through LLMs virtual interactions: A case study with GPT-3.5. 2023 IEEE International Conference on Bioinformatics and Biomedicine (BIBM).

Uluslu, A. Y., Michail, A., & Clematide, S. (2024). Utilizing large language models to identify evidence of suicidality risk through analysis of emotionally charged posts. Proceedings of the 9th Workshop on Computational Linguistics and Clinical Psychology.

Wang, X., Liu, K., & Wang, C. (2023). Knowledge-enhanced pre-training large language model for depression diagnosis and treatment. 2023 IEEE 9th International Conference on Cloud Computing and Intelligent Systems.

Xu, X., Yao, B., Dong, Y., Gabriel, S., Yu, H., Hendler, J., ... & Wang, D. (2024). Mental-llm: Leveraging large language models for mental health prediction via online text data. Proceedings of the ACM on Interactive, Mobile, Wearable and Ubiquitous Technologies, 8(1), 1-32.

Yang, K., Zhang, T., Kuang, Z., Xie, Q., Huang, J., & Ananiadou, S. (2024). MentaLLaMA: interpretable mental health analysis on social media with large language models. Proceedings of the ACM on Web Conference 2024.

Zhang, T., Teng, S., Jia, H., & D'Alfonso, S. (2024a). Leveraging LLMs to predict affective states via smartphone sensor features. Companion of the 2024 on ACM International Joint Conference on Pervasive and Ubiquitous Computing.

Zhang, T., Yang, K., Ji, S., Liu, B., Xie, Q., & Ananiadou, S. (2024b). SuicidEmoji: Derived Emoji dataset and tasks for suicide-related social content. Proceedings of the 47th International ACM SIGIR Conference on Research and Development in Information Retrieval.

Zhou, W., Prater, L. C., Goldstein, E. V., & Mooney, S. J. (2023). Identifying rare circumstances preceding female firearm suicides: Validating a large language model approach. JMIR mental health, 10(1), e49359.

Zhu, J., Xu, A., Tan, M., & Yang, M. (2024, March). XinHai@ CLPsych 2024 Shared Task: Prompting Healthcare-oriented LLMs for Evidence Highlighting in Posts with Suicide Risk. Proceedings of the 9th Workshop on Computational Linguistics and Clinical Psychology.

GLM as Therapeutic Tools

Alanezi, F. (2024). Assessing the effectiveness of GPT-3.5 in delivering mental health support: A qualitative study. Journal of Multidisciplinary Healthcare, 461-471.

Alessa, A., & Al-Khalifa, H. (2023, July). Towards designing a GPT-3.5 conversational companion for elderly people. Proceedings of the 16th international conference on Pervasive technologies related to assistive environments.

Berrezueta-Guzman, S., Kandil, M., Martín-Ruiz, M. L., Pau de la Cruz, I., & Krusche, S. (2024). Future of ADHD care: Evaluating the efficacy of GPT-3.5 in therapy enhancement. Healthcare (Basel), 12(6).

Brocki, L., Dyer, G. C., G_adka, A., & Chung, N. C. (2023). Deep learning mental health dialogue system. 2023 IEEE International Conference on Big Data and Smart Computing.

De Freitas, J., U_uralp, A. K., O_uz_U_uralp, Z., & Puntoni, S. (2024). Chatbots and mental health: Insights into the safety of GAI. Journal of Consumer Psychology, 34(3), 481-491.

Dongre, P. (2024, May). Physiology-Driven Empathic Large Language Models (EmLLMs) for Mental Health Support. Extended Abstracts of the CHI Conference on Human Factors in Computing Systems.

Herencia López-Menchero, A. (2024). Analysis of the Transformer Architecture and application on a Large Language Model for mental health counseling. [Master Thesis, Complutense University of Madrid]. https://hdl.handle.net/20.500.14352/106894

Heston, T. F. (2023). Safety of Large Language Models in Addressing Depression. Cureus, 15(12), e50729. https://doi.org/10.7759/cureus.50729

Kumar, H., Wang, Y., Shi, J., Musabirov, I., Farb, N. A., & Williams, J. J. (2023). Exploring the use of large language models for improving the awareness of mindfulness. Extended Abstracts of the 2023 CHI Conference on Human Factors in Computing Systems.

Ma, Z., Mei, Y., & Su, Z. (2023). Understanding the benefits and challenges of using large language model-based conversational agents for mental well-being support. AMIA Annual Symposium Proceedings.

Ma, Z., Mei, Y., Long, Y., Su, Z., & Gajos, K. Z. (2024, May). Evaluating the experience of LGBTQ+ people using large language model based chatbots for

mental health support. Proceedings of the CHI Conference on Human Factors in Computing Systems.

Najarro, L. A., Lee, Y., Toshnazarov, K. E., Jang, Y., Kim, H., & Noh, Y. (2023). WMGPT: Towards 24/7 online prime counseling with GPT-3.5. Adjunct Proceedings of the 2023 ACM International Joint Conference on Pervasive and Ubiquitous Computing & the 2023 ACM International Symposium on Wearable Computing.

Sharma, A., Rushton, K., Lin, I. W., Nguyen, T., & Althoff, T. (2024). Facilitating self-guided mental health interventions through human-language model interaction: A case study of cognitive restructuring. Proceedings of the CHI Conference on Human Factors in Computing Systems.

Singh, A., Ehtesham, A., Mahmud, S., & Kim, J. H. (2024). Revolutionizing mental health care through langchain: A journey with a large language model. 2024 IEEE 14th Annual Computing and Communication Workshop and Conference.

Vakayil, S., Juliet, D. S., & Vakayil, S. (2024). RAG-based LLM chatbot using Llama-2. 2024 7th International Conference on Devices, Circuits and Systems.

Vowels, L. M., Francois-Walcott, R. R., & Darwiche, J. (2024). AI in relationship counselling: Evaluating GPT-3.5’s therapeutic capabilities in providing relationship advice. Computers in Human Behavior: Artificial Humans, 100078.

Wu, R., Yu, C., Pan, X., Liu, Y., Zhang, N., Fu, Y., Wang, Y., Zheng, Z., Chen, L., Jiang, Q., Xu, X., & Shi, Y. (2024). MindShift: Leveraging large language models for mental-states-based problematic smartphone use intervention.

Proceedings of the 2024 CHI Conference on Human Factors in Computing Systems.

Yahagi, M., Hiruta, R., Miyauchi, C., Tanaka, S., Taguchi, A., & Yaguchi, Y. (2024). Comparison of Conventional Anesthesia Nurse Education and an Artificial Intelligence Chatbot (GPT-3.5) Intervention on Preoperative Anxiety: A randomized controlled Trial. Journal of PeriAnesthesia Nursing.

Yu, H., & McGuinness, S. (2024). An experimental study of integrating fine-tuned LLMs and prompts for enhancing mental health support chatbot system. Journal of Medical Artificial Intelligence, 1-16.

GLM for Supporting Clinicians and Mental Health Professionals

Adhikary, P. K., Srivastava, A., Kumar, S., Singh, S. M., Manuja, P., Gopinath, J. K., Krishnan, V., Gupta, K., Deb, K. S., & Chakraborty, T. (2024). Exploring the efficacy of large language models in summarizing mental health counseling sessions: Benchmark study. JMIR Mental Health, 11.

Bird, J. J., & Lotfi, A. (2023). Generative Transformer chatbots for Mental Health Support: A Study on Depression and Anxiety Proceedings of the 16th International Conference on PErvasive Technologies Related to Assistive Environments, Corfu, Greece. https://doi.org/10.1145/3594806.3596520

Dergaa, I., Fekih-Romdhane, F., Hallit, S., Loch, A. A., Glenn, J. M., Fessi, M. S., Ben Aissa, M., Souissi, N., Guelmami, N., & Swed, S. (2024). GPT-3.5 is not

ready yet for use in providing mental health assessment and interventions. Frontiers in Psychiatry, 14, 1277756.

D’Souza, R. F., Amanullah, S., Mathew, M., & Surapaneni, K. M. (2023). Appraising the performance of GPT-3.5 in psychiatry using 100 clinical case vignettes. Asian Journal of Psychiatry, 89, 103770.

Englhardt, Z., Ma, C., Morris, M. E., Chang, C. C., Xu, X. O., Qin, L., ... & Iyer, V. (2024). From classification to clinical insights: Towards analyzing and reasoning about mobile and behavioral health data with large language models. Proceedings of the ACM on Interactive, Mobile, Wearable and Ubiquitous Technologies, 8(2), 1-25.

Elyoseph, Z., & Levkovich, I. (2024). Comparing the perspectives of GAI, mental health experts, and the general public on schizophrenia recovery: Case vignette study. JMIR Mental Health, 11, e53043.

Elyoseph, Z., Levkovich, I., & Shinan-Altman, S. (2024). Assessing prognosis in depression: Comparing perspectives of AI models, mental health professionals and the general public. Family Medicine and Community Health, 12(Suppl 1).

Furukawa, T. A., Iwata, S., Horikoshi, M., Sakata, M., Toyomoto, R., Luo, Y., Tajika, A., Kudo, N., & Aramaki, E. (2023). Harnessing AI to optimize thought records and facilitate cognitive restructuring in smartphone CBT: An exploratory study. Cognitive Therapy and Research, 47(6), 887-893.

Giorgi, S., Isman, K., Liu, T., Fried, Z., Sedoc, J., & Curtis, B. (2024). Evaluating GAI responses to real-world drug-related questions. Psychiatry Research, 339, 116058.

Hadar-Shoval, D., Elyoseph, Z., & Lvovsky, M. (2023). The plasticity of GPT-3.5’s mentalizing abilities: Personalization for personality structures. Frontiers in Psychiatry, 14, 1234397.

Hsieh, L. H., Liao, W. C., & Liu, E. Y. (2024). Feasibility assessment of using GPT-3.5 for training case conceptualization skills in psychological counseling. Computers in Human Behavior: Artificial Humans, 2(2), 100083.

Hedderich, M. A., Bazarova, N. N., Zou, W., Shim, R., Ma, X., & Yang, Q. (2024). A piece of theatre: Investigating how teachers design LLM chatbots to assist adolescent cyberbullying education. Proceedings of the 2024 CHI Conference on Human Factors in Computing Systems.

Hodson, N., & Williamson, S. (2024). Can Large Language Models Replace Therapists? Evaluating Performance at Simple Cognitive Behavioral Therapy Tasks. JMIR AI, 3(1), e52500.

Hu, Z., Hou, H., & Ni, S. (2024, June). Grow with your AI buddy: Designing an LLMs-based conversational agent for the measurement and cultivation of children's mental resilience. Proceedings of the 23rd Annual ACM Interaction Design and Children Conference.

Hwang, G., Lee, D. Y., Seol, S., Jung, J., Choi, Y., Her, E. S., ... & Park, R. W. (2024). Assessing the potential of GPT-3.5 for psychodynamic formulations in psychiatry: An exploratory study. Psychiatry Research, 331, 115655.

James, L. J., Genga, L., Montagne, B., Hagenaars, M., & Van Gorp, P. (2024). Caregiver's evaluation of LLM-generated treatment goals for patients with severe mental illnesses. Proceedings of the 17th International Conference on PErvasive Technologies Related to Assistive Environments.

Kim, T., Bae, S., Kim, H. A., Lee, S. W., Hong, H., Yang, C., & Kim, Y. H. (2024). MindfulDiary: Harnessing large language model to support psychiatric patients' journaling. Proceedings of the CHI Conference on Human Factors in Computing Systems.

Levkovich, I., & Elyoseph, Z. (2023). Suicide risk assessments through the eyes of GPT-3.5 versus GPT-4: Vignette Study. JMIR Mental Health, 10, e51232. https://doi.org/10.2196/51232

Liu, Y., Ding, X., Peng, S., & Zhang, C. (2024). Leveraging GPT-3.5 to optimize depression intervention through explainable deep learning. Frontiers in Psychiatry, 15, 1383648.

Maurya, R. K., Montesinos, S., Bogomaz, M., & DeDiego, A. C. (2023). Assessing the use of GPT-3.5 as a psychoeducational tool for mental health practice. Counselling and Psychotherapy Research.

Meyer, S., & Elsweiler, D. (2024). "You tell me": A dataset of GPT-4-based behaviour change support conversations. Proceedings of the 2024 Conference on Human Information Interaction and Retrieval.

Perlis, R. H., Goldberg, J. F., Ostacher, M. J., & Schneck, C. D. (2024). Clinical decision support for bipolar depression using large language models. Neuropsychopharmacology, 49(9), 1412-1416. https://doi.org/10.1038/s41386-024-01841-2

Smith, A., Hachen, S., Schleifer, R., Bhugra, D., Buadze, A., & Liebrenz, M. (2023). Old dog, new tricks? Exploring the potential functionalities of GPT-3.5 in supporting educational methods in social psychiatry. International Journal of Social Psychiatry, 69(8), 1882-1889.

Wu, Y., Mao, K., Zhang, Y., & Chen, J. (2024). CALLM: Enhancing clinical interview analysis through data augmentation with large language models. IEEE Journal of Biomedical and Health Informatics.
