# Supplementary material for: The Application and Ethical Implication of Generative AI in Mental Health: Systematic Review
Source: JMIR Ment Health. 2025 Jun 27;12:e70610. doi: 10.2196/70610 (PMC12254713; doi:10.2196/70610)
Supplement: Multimedia Appendix 4 [file mental_v12i1e70610_app4.pdf]

MI-CLAIM-GEN Checklist for generative AI clinical studies

Section	Checklist
Part 1: Study design	1.1 The clinical problem in which the model will be employed is clearly detailed in the paper
	1.2 The research question is clearly stated
	1.3 All cohort selection criteria and study design are detailed in such a way that they can be reproduced by an external researcher
	1.4 Identify whether the output data type categorical, continuous, or unstructured
	1.5 The characteristics of the cohorts are detailed in the text and are shown to be representative of real-world clinical settings
Part 2: Resources and optimization	2.1 Model/application components are clearly detailed including: base model(s) used, embedding model(s), retrieval model(s), and other auxiliary models or tools
	2.2 The origin of all data sources for model training, finetuning, or inference is described and the original format is detailed in the paper
	2.3 All data preprocessing for model training, finetuning, or inference is described, including appropriate randomization and other transformations
	2.4 The independence between training, validation (including for prompt engineering), and test sets has been described, and data are split at the patient level
Parts 3: Model performance and evaluation	3.1 The state-of-the-art solution used as a baseline for comparison has been identified and detailed
	3.2 The performance comparison between the baseline and the proposed model is presented with the appropriate statistical significance
	3.3 Identify what evaluation(s) were performed, and provide clear justifications for the primary metrics used for each evaluation; describe whether overlap accuracy, semantic accuracy, and/or clinical utility were assessed
	3.4 If applicable, details on human evaluation are described, including any evaluation guidelines, level of experience of evaluators, inter-reviewer scores, etc
Part 4: Model examination	4.1 Relevant interpretability techniques, error analysis, and/or other approaches are applied to demonstrate an absence of unreasonable risk and brittleness, including a low

	risk of catastrophic and especially undetected failure
	4.2 A discussion of the risk revealed by the examination results is presented with respect to model/algorithm performance
	4.3 Describe step(s) taken to discuss, identify, and/or mitigate model biases, privacy and security concerns, and other potential harms
	4.4 A discussion and/or assessment of relevant distribution shifts and their impact on the model's performance has been provided
	4.5 Recommendations or discussion of post-deployment evaluation have been provided
Part 5: Reproducibility; data and model transparency	5.1 Choose appropriate tier:<br>Tier 1: complete sharing of the code and data, including all prompts tested, hyperparameters used, software dependencies, model versions, and compute requirements<br>Tier 2A: complete sharing of the code with synthetic data provided<br>Tier 2B: complete sharing of the code<br>Tier 3: no sharing of code or data
	5.2 A clinical model card is included summarizing the model capabilities, intended use, descriptions of any dataset or other integrations, limitations, potential biases, and risks
	5.3 If applicable, model weights are released to a secure repository with appropriate use agreements

Reference:
Miao, B. Y., Chen, I. Y., Williams, C. Y., Davidson, J., Garcia-Agundez, A., Sun, S., ... & Sushil, M. (2025). The MI-CLAIM-GEN checklist for generative artificial intelligence in health. *Nature Medicine*, 1-5.
