# Supplementary material for: The Application and Ethical Implication of Generative AI in Mental Health: Systematic Review
Source: JMIR Ment Health. 2025 Jun 27;12:e70610. doi: 10.2196/70610 (PMC12254713; doi:10.2196/70610)
Supplement: Multimedia Appendix 5 [file mental_v12i1e70610_app5.pdf]

Supplementary Table 3. Summary of Studies on GAI for Mental Health Diagnosis and Assessment.

	Mental Health Issue	GAI Used	Data Source	Key Findings
Alhamed et al., 2024 [1]	Suicide Risk	LLaMA-2	Reddit Posts	Combining LLMs with lexicon-based approaches for precise suicide risk evidence extraction from social media posts.
Bauer et al., 2024 [2]	Suicide Risk	GPT-4	Reddit Posts	Analyzing Reddit posts with LLMs to identify suicidality-linked linguistic patterns that support suicide theories and improve risk assessment.
Chen et al., 2024a [3]	Suicide Risk	LLaMA-2	Reddit Posts	Extracting evidence supporting suicide risk assessment effectively with LLMs.
Chen et al., 2024b [4]	Depression	GPT-3.5, GPT-4	Clinical Interviews	Enhancing the SEGA model with LLM-guided data augmentation improves depression detection accuracy in clinical interviews.
Danner et al., 2023 [5]	Depression	GPT-3.5, GPT-4	Clinical Interviews	Outperforming other models in detecting depression with high accuracy using GPT-3.5-based models.
D'Souza et al., 2023 [6]	Multiple mental disorders	GPT-3.5	100 Clinical Case Vignettes	Performing well in diagnosing psychiatric conditions with GPT-3.5, receiving mostly Grade A and B ratings.
Elyoseph & Levkovich, 2023 [7]	Suicide Risk	GPT-3.5	Text Vignette	Underestimating the risk of suicide attempts compared to professionals with ChatGPT, particularly in cases of high burdensomeness and belongingness, suggesting caution for suicide risk assessment.
Englhardt et al., 2024 [8]	Depression, Anxiety	GPT-4, GPT-3.5, PaLM-2	Multi-sensory Data	Generating clinical insights from multi-sensor data with LLMs to support collaborative patient-clinician exploration, recognized by mental health professionals.
Gargari et al., 2024 [9]	Multiple mental disorders	GPT-3.5, GPT-4, Aya, Nemotron	DSM-5 Clinical Cases	Demonstrating superior diagnostic accuracy and reasoning quality in psychiatric assessments with GPT-3.5 and GPT-4 compared to other models.
Hayati et al., 2022 [10]	Depression	GPT-3	Malay dialectal speeches	Showing promise in detecting depression from dialectal Malay speech, especially for the Kuala Lumpur dialect, with GPT-3.

Heinz et al., 2023 [11]	Multiple mental disorders	GPT-3	Clinical vignettes	Demonstrating varying performance across different diagnoses with excellent accuracy for ADHD, PTSD, and alcohol use disorder, but poorer performance for bipolar disorder and somatic symptom disorder.
Hu et al., 2024 [12]	Depression	GPT-4o	Multimodal Data	Improving mental health prediction accuracy using multimodal data, particularly EEG, and achieving better results with few-shot learning than zero-shot learning.
Hur et al., 2024 [13]	Depression	GPT-3.5	Written responses of Participants	Predicting changes in depression over three weeks with human-rated and ChatGPT-rated sentiment, while LIWC shows less effectiveness.
Kim et al., 2024a [14]	OCD	GPT-4, Gemini Pro, LLaMA-3	Clinical vignettes	Outperforming mental health professionals in accurately diagnosing OCD with LLMs, highlighting their potential in mental health care.
Lee et al., 2024 [15]	Suicidal Ideation	GPT-4	Brightside Intake	Approaching clinician-level performance in predicting suicidal ideation with GPT-4, producing quicker results than clinicians, suggesting its potential to augment human assessments.
Levkovich & Elyoseph, 2023 [16]	Suicide Risk	GPT-3.5, GPT-4	Text Vignette	Matching professional assessments closely in suicide attempt evaluation with GPT-4, while GPT-3.5 consistently underestimates suicide risk.
Li et al., 2024 [17]	Depression	GPT-3.5	Reddit Posts	Improving mental health detection accuracy on social media with MAIMS, providing transparent and scientifically grounded analysis.
Mazumdar et al., 2023 [18]	Depression, Burdensomeness	GPT-3	Reddit Posts	Enhancing classification accuracy and coherence of explanations in mental health detection by fine-tuning GPT-3 with contextual embeddings.
Ni et al., 2023 [19]	Emotional Regulation	GPT-3.5	Adolescent Reports	Facilitating psychoecological data collection using Beatrice, with high usability scores, simplifying questionnaire deployment and providing an interactive user experience.

Ohse et al., 2024 [20]	Depression	GPT-3.5, GPT-4, LLaMA-2	Clinical Interviews	Achieving the highest accuracy in depression classification with GPT-4 and a strong correlation in symptom severity estimation, especially with fine-tuning.
Pugh et al., 2024 [21]	Schizophrenia	GPT-3.5, GPT-4, LLaMA-3	Speech Transcripts	Predicting human ratings of coherence, content, and tangentiality with moderate accuracy, though inconsistencies in LLM-generated ratings are observed, influenced by temperature parameters.
Radwan et al., 2024 [22]	Stress	GPT-3	Reddit Posts	Identifying stress-related indicators in social media posts effectively by combining GPT-3 embeddings with machine learning models like SVM.
Saleem & Kim, 2024 [23]	Stress	GPT-3.5	Reddit Posts	Improving stress detection by 3% with intent-aware data augmentation techniques using same and opposite intents.
Shin et al., 2024 [24]	Depression	GPT-3.5, GPT-4	Diary Texts	Achieving 90.2% accuracy with GPT-3.5 fine-tuning and perfect recall with GPT-4 in identifying depressive cases, highlighting clinical potential.
Shinan-Altman et al., 2024 [25]	Suicide Risk	GPT-3.5, GPT-4	Text Vignette	Assigning higher severity ratings with GPT-4 in assessing the relationship between depression, access to weapons, and suicide risk, compared to GPT-3.5.
Singh et al., 2024 [26]	Suicidal Ideation	Mixtral-7bx8, Tulu-2-DPO-70B	Reddit Posts	Improving evidence extraction performance with chain-of-thought prompting, especially in few-shot learning, for mental health monitoring.
Soun & Nair, 2024 [27]	Suicide Risk, Stress	GPT-3.5	Reddit Posts	Demonstrating bias in GPT-3.5 performance, favoring young females (18–30), likely due to training data imbalances.
Stern et al., 2024 [28]	Suicide Risk	LLaMA, WizardLM, SpeechlessLM	Reddit Posts	Generating natural language explanations for suicide risk assessment in social media posts using LLMs, showing moderate alignment with expert evaluations but challenges in interpretability.
Tao et al., 2023 [29]	Anxiety, Depression	GPT-3.5	Speech Data	Classifying anxiety and depression effectively with ChatGPT, especially when combined with additional features like speech rhythm and speed.

Uluslu et al., 2024 [30]	Suicide RIsk	Mistral-7B	Reddit Posts	Identifying suicide risk evidence from emotionally charged social media posts effectively with LLM and RAG combination, aligning with expert assessments.
Wang et al., 2023 [31]	Depression	GPT-3	Medical Dialogue	Improving diagnostic accuracy and safety in depression treatment dialogues with a knowledge-enhanced pre-training approach for LLMs.
Xu et al., 2024 [32]	Depression, Stress, Suicide Risk	GPT-3.5, GPT-4, FLAN-T5, Alpaca	Reddit Posts	Boosting model performance in mental health prediction tasks with instruction fine-tuning across datasets, with models like Mental-Alpaca outperforming GPT-4 in some tasks.
Yang et al., 2024 [33]	Multiple mental disorders	LLaMA-2	Reddit, Twitter, SMS texts	Enabling MentaLLaMA to perform interpretable mental health analysis on social media with near state-of-the-art accuracy through task-specific, instruction-based fine-tuning.
Zhang et al., 2024a [34]	Affective States	Gemini-1.5 Pro	Smartphone sensor data	Demonstrating promising results in predicting emotional states with LLMs, especially in few-shot learning, connecting smartphone data with affect.
Zhang et al., 2024b [35]	Suicidal Ideation	Falcon, GPT-3.5, LLaMA	Reddit Posts	Enhancing suicide ideation detection by incorporating emoji features, though models struggle with accurate emoji prediction due to ambiguity.
Zhou et al., 2023 [36]	Suicide (Female Firearm Suicide)	FLAN-UL2	National Violent Death Reporting System	Outperforming traditional NLP approaches in identifying rare circumstances preceding female firearm suicides, achieving higher accuracy with an LLM.
Zhu et al., 2024 [37]	Suicidal Ideation	XinHai	Reddit Posts	Improving recall and precision in identifying suicide-related evidence and generating summaries in online discussions with fine-tuned LLMs, especially those with healthcare knowledge.

Reference

1. Alhamed F, Ive J, Specia L, editors. Using Large Language Models (LLMs) to Extract Evidence from Pre-Annotated Social Media Data. Proceedings of the 9th Workshop on Computational Linguistics and Clinical Psychology (CLPsych 2024); 2024.
2. Bauer B, Norel R, Leow A, Abi Rached Z, Wen B, Cecchi G. Using Large Language Models to Understand Suicidality in a Social Media–Based Taxonomy of Mental Health Disorders: Linguistic Analysis of Reddit Posts. JMIR Mental Health. 2024;11. doi: 10.2196/57234.
3. Chen J, Nguyen V, Dai X, Molla D, Paris C, Karimi S, editors. Exploring Instructive Prompts for Large Language Models in the Extraction of Evidence for Supporting Assigned Suicidal Risk Levels. Proceedings of the 9th Workshop on Computational Linguistics and Clinical Psychology (CLPsych 2024); 2024.
4. Chen Z, Deng J, Zhou J, Wu J, Qian T, Huang M, editors. Depression detection in clinical interviews with LLM-empowered structural element graph. Proceedings of the 2024 Conference of the North American Chapter of the Association for Computational Linguistics: Human Language Technologies (Volume 1: Long Papers); 2024.
5. Danner M, Hadzic B, Gerhardt S, Ludwig S, Uslu I, Shao P, et al., editors. Advancing mental health diagnostics: GPT-based method for depression detection. 2023 62nd Annual Conference of the Society of Instrument and Control Engineers (SICE); 2023: IEEE.
6. D'Souza RF, Amanullah S, Mathew M, Surapaneni KM. Appraising the performance of ChatGPT in psychiatry using 100 clinical case vignettes. Asian Journal of Psychiatry. 2023;89:103770.
7. Elyoseph Z, Levkovich I. Beyond human expertise: the promise and limitations of ChatGPT in suicide risk assessment. Front Psychiatry. 2023;14:1213141. PMID: 37593450. doi: 10.3389/fpsyt.2023.1213141.
8. Englhardt Z, Ma C, Morris ME, Chang C-C, Xu XO, Qin L, et al. From Classification to Clinical Insights: Towards Analyzing and Reasoning About Mobile and Behavioral Health Data With Large Language Models. Proc ACM Interact Mob Wearable Ubiquitous Technol. 2024;8(2):Article 56. doi: 10.1145/3659604.
9. Gargari OK, Fatehi F, Mohammadi I, Firouzabadi SR, Shafiee A, Habibi G. Diagnostic accuracy of large language models in psychiatry. Asian Journal of Psychiatry. 2024;100. doi: 10.1016/j.ajp.2024.104168.
10. Hayati MFM, Ali MAM, Rosli ANM, editors. Depression Detection on Malay Dialects Using GPT-3. 7th IEEE-EMBS Conference on Biomedical Engineering and Sciences, IECBES 2022 - Proceedings; 2022.

11. Heinz MV, Bhattacharya S, Trudeau B, Quist R, Song SH, Lee CM, et al. Testing domain knowledge and risk of bias of a large-scale general artificial intelligence model in mental health. Digit Health. 2023 Jan-Dec;9:20552076231170499. PMID: 37101589. doi: 10.1177/20552076231170499.
12. Hu Y, Zhang S, Dang T, Jia H, Salim FD, Hu W, et al. Exploring Large-Scale Language Models to Evaluate EEG-Based Multimodal Data for Mental Health. Companion of the 2024 on ACM International Joint Conference on Pervasive and Ubiquitous Computing; Melbourne VIC, Australia: Association for Computing Machinery; 2024. p. 412–7.
13. Hur JK, Heffner J, Feng GW, Joormann J, Rutledge RB. Language sentiment predicts changes in depressive symptoms. Proc Natl Acad Sci U S A. 2024 Sep 24;121(39):e2321321121. PMID: 39284070. doi: 10.1073/pnas.2321321121.
14. Kim J, Leonte KG, Chen ML, Torous JB, Linos E, Pinto A, et al. Large language models outperform mental and medical health care professionals in identifying obsessive-compulsive disorder. NPJ Digit Med. 2024 Jul 19;7(1):193. PMID: 39030292. doi: 10.1038/s41746-024-01181-x.
15. Lee C, Mohebbi M, O'Callaghan E, Winsberg M. Large Language Models Versus Expert Clinicians in Crisis Prediction Among Telemental Health Patients: Comparative Study. JMIR Ment Health. 2024 Aug 2;11:e58129. PMID: 38876484. doi: 10.2196/58129.
16. Levkovich I, Elyoseph Z. Suicide Risk Assessments Through the Eyes of ChatGPT-3.5 Versus ChatGPT-4: Vignette Study. JMIR Ment Health. 2023 Sep 20;10:e51232. PMID: 37728984. doi: 10.2196/51232.
17. Li W, Zhu Y, Lin X, Li M, Jiang Z, Zeng Z. Zero-shot Explainable Mental Health Analysis on Social Media by Incorporating Mental Scales. Companion Proceedings of the ACM Web Conference 2024; Singapore, Singapore: Association for Computing Machinery; 2024. p. 959–62.
18. Mazumdar H, Chakraborty C, Sathvik M, Panigrahi PK. GPTFX: A novel GPT-3 based framework for mental health detection and explanations. IEEE Journal of Biomedical and Health Informatics. 2023.
19. Ni Y, Chen Y, Ding R, Ni S. Beatrice: A Chatbot for Collecting Psychoecological Data and Providing QA Capabilities. Proceedings of the 16th International Conference on PErvasive Technologies Related to Assistive Environments; Corfu, Greece: Association for Computing Machinery; 2023. p. 429–35.
20. Ohse J, Hadžić B, Mohammed P, Peperkorn N, Danner M, Yorita A, et al. Zero-Shot Strike: Testing the generalisation capabilities of out-of-the-box LLM models for depression detection. Computer Speech & Language. 2024;88:101663.

21. Pugh SL, Chandler C, Cohen AS, Diaz-Asper C, Elvevåg B, Foltz PW. Assessing dimensions of thought disorder with large language models: The tradeoff of accuracy and consistency. Psychiatry Research. 2024;341. doi: 10.1016/j.psychres.2024.116119.
22. Radwan A, Amarneh M, Alawneh H, Ashqar HI, AlSobeh A, Magableh AAAR. Predictive analytics in mental health leveraging llm embeddings and machine learning models for social media analysis. International Journal of Web Services Research (IJWSR). 2024;21(1):1-22.
23. Saleem M, Kim J. Intent aware data augmentation by leveraging generative AI for stress detection in social media texts. PeerJ Computer Science. 2024;10:e2156.
24. Shin D, Kim H, Lee S, Cho Y, Jung W. Using Large Language Models to Detect Depression From User-Generated Diary Text Data as a Novel Approach in Digital Mental Health Screening: Instrument Validation Study. J Med Internet Res. 2024 Sep 18;26:e54617. PMID: 39292502. doi: 10.2196/54617.
25. Shinan-Altman S, Elyoseph Z, Levkovich I. The impact of history of depression and access to weapons on suicide risk assessment: a comparison of ChatGPT-3.5 and ChatGPT-4. PeerJ. 2024;12:e17468. PMID: 38827287. doi: 10.7717/peerj.17468.
26. Singh LG, Mao J, Mutalik R, Middleton S, editors. Extracting and Summarizing Evidence of Suicidal Ideation in Social Media Contents Using Large Language Models. Proceedings of the 9th Workshop on Computational Linguistics and Clinical Psychology (CLPsych 2024); 2024.
27. Soun RS, Nair A. ChatGPT for Mental Health Applications: A study on biases. Proceedings of the Third International Conference on AI-ML Systems; Bangalore, India: Association for Computing Machinery; 2024. p. Article 38.
28. Stern W, Goh SJ, Nur N, Aragon PJ, Mercer T, Bhattacharyya S, et al., editors. Natural Language Explanations for Suicide Risk Classification Using Large Language Models. ML4CMH@ AAAI; 2024.
29. Tao Y, Yang M, Shen H, Yang Z, Weng Z, Hu B, editors. Classifying anxiety and depression through LLMs virtual interactions: A case study with ChatGPT. 2023 IEEE International Conference on Bioinformatics and Biomedicine (BIBM); 2023: IEEE.
30. Uluslu AY, Michail A, Clematide S, editors. Utilizing large language models to identify evidence of suicidality risk through analysis of emotionally charged posts. 2024: Association for Computational Linguistics.
31. Wang X, Liu K, Wang C, editors. Knowledge-enhanced pre-training large language model for depression diagnosis and treatment. 2023 IEEE 9th International Conference on Cloud Computing and Intelligent Systems (CCIS); 2023: IEEE.
32. Xu X, Yao B, Dong Y, Gabriel S, Yu H, Hendler J, et al. Mental-LLM: Leveraging Large Language Models for Mental Health Prediction via Online Text Data. Proc ACM Interact Mob Wearable Ubiquitous Technol. 2024;8(1):Article 31. doi: 10.1145/3643540.

33. Yang K, Zhang T, Kuang Z, Xie Q, Huang J, Ananiadou S. MentaLLaMA: Interpretable Mental Health Analysis on Social Media with Large Language Models. Proceedings of the ACM Web Conference 2024; Singapore, Singapore: Association for Computing Machinery; 2024. p. 4489–500.
34. Zhang T, Teng S, Jia H, D'Alfonso S. Leveraging LLMs to Predict Affective States via Smartphone Sensor Features. Companion of the 2024 on ACM International Joint Conference on Pervasive and Ubiquitous Computing; Melbourne VIC, Australia: Association for Computing Machinery; 2024. p. 709–16.
35. Zhang T, Yang K, Ji S, Liu B, Xie Q, Ananiadou S. SuicidEmoji: Derived Emoji Dataset and Tasks for Suicide-Related Social Content. Proceedings of the 47th International ACM SIGIR Conference on Research and Development in Information Retrieval; Washington DC, USA: Association for Computing Machinery; 2024. p. 1136–41.
36. Zhou W, Prater LC, Goldstein EV, Mooney SJ. Identifying Rare Circumstances Preceding Female Firearm Suicides: Validating A Large Language Model Approach. JMIR Ment Health. 2023 Oct 17;10:e49359. PMID: 37847549. doi: 10.2196/49359.
37. Zhu J, Xu A, Tan M, Yang M, editors. Xinhai@ clpsych 2024 shared task: Prompting healthcare-oriented llms for evidence highlighting in posts with suicide risk. Proceedings of the 9th Workshop on Computational Linguistics and Clinical Psychology (CLPsych 2024); 2024.
