# Supplementary material for: The Application and Ethical Implication of Generative AI in Mental Health: Systematic Review
Source: JMIR Ment Health. 2025 Jun 27;12:e70610. doi: 10.2196/70610 (PMC12254713; doi:10.2196/70610)
Supplement: Multimedia Appendix 6 [file mental_v12i1e70610_app6.pdf]

Supplementary Table 4. List of datasets used in studies on GLMs for mental health diagnosis and assessment.

| Dataset | Sample Size | Population | Modalities | Mental Health Issues |
|---|---|---|---|---|
| 100 Cases in Psychiatry [1] | 100 cases | N/A | Text (clinical vignettes) | Various psychiatric disorders |
| Beatrice-ILD [2] | 317 | Chinese adolescents | Daily self-reports | Emotion regulation |
| Brightside SI Dataset [3] | 460 | Telemental health patients | Transcripts | Suicidal ideation with a plan |
| CAMS [4] | 5,051 posts | Reddit users | Text (posts) | Depression, suicidal tendencies |
| Clinical Vignettes Dataset [5] | 59 cases | N/A | Text (clinical vignettes) | Various psychiatric disorders |
| CLPsych 2015 [6] | 1,746 | Twitter users | Text (posts) | Depression, Post-Traumatic Stress Disorder (PTSD) |
| CLPsych 2024 [7] | 125 users | Reddit users | Text (posts) | Suicidality |
| CSSRS-Suicide [8] | 2,181 users | Suicidal ideation users | Text (posts) | Suicide ideation, behavior, attempt |
| DAIC-WOZ [9] | 189 | General public | Transcripts, audios, videos | Anxiety, depression and PTSD |

| | | | | |
|---|---|---|---|---|
| Depression Language Dataset [2] | 467 | General population | Transcripts | Depression |
| Depression_Reddit [10] | 800 | Reddit users | Text (posts) | Depression |
| DepSeverity [11] | 3,553 posts | Reddit users | Text (posts) | Depression |
| DREADDIT [12] | 190,000 | Reddit users | Text (posts) | Stress, anxiety, PTSD |
| DSM-5 Clinical Cases [13] | 20 cases | N/A | Text (clinical vignettes) | Various psychiatric disorders |
| EATD [14] | 162 | University students and teachers | Transcripts, audios | Depression |
| Emotional Diary Text Data [15] | 428 diaries from 91 participants | General public | Text (diaries) | Depression, suicide risk |
| Extended-DAIC [16] | 275 | General public | Transcripts, audios, videos | Depression, PTSD |
| GLOBEM [17] | 705 person-years, 497 unique participants | University students | Location, phone usage, Bluetooth, call logs, physical activity, sleep data | Depression, anxiety |
| IRF [18] | 3,522 | Reddit users | Text (posts) | TBE, PBU |
| LUMED-2 [19] | 13(6 females, 7 males) | General population | EEG, GSR, facial expressions | Emotion recognition |
| Malay Dialect Depression Dataset [20] | 53 | Malaysian adults | Transcripts, audios | Depression |

| | | | | |
|---|---|---|---|---|
| MedDialog [21] | 3.4 million conversations | General population in China | Text | Various psychiatric disorders |
| MODMA [22] | 108 for EEG, 52 for spoken language | Clinically diagnosed depression patients and healthy controls | EEG, Audio | Major Depressive Disorder (MDD) |
| NVDRS Female Firearm Suicide Dataset [23] | 1,462 | Female firearm suicide decedents | Text | Suicide risk factors |
| OCD Vignette Dataset [24] | 26 vignettes | Patients with psychiatric conditions | Text (clinical vignettes) | Various psychiatric disorders |
| PME4 [25] | 11 | Acting students with prior acting experience | EEG, EMG, audio, video | Emotion recognition |
| Reddit Dataset (2022 & 2010) [26] | 2.9 million posts | Reddit users | Text (posts) | Suicidal ideation and various psychiatric disorders |
| Reddit Stress Analysis Dataset [27] | 3,553 posts | General public | Text (posts) | Stress, depression, anxiety, PTSD, substance abuse |
| Red-Sam [28] | 16,632 | Reddit users | Text (posts) | Depression |

| | | | | |
|---|---|---|---|---|
| SAD [29] | 6,850 | General public | Text | Stress |
| SDCNL [30] | 1,895 | Reddit users | Text (posts) | Depression, suicidal ideation |
| Suicide Risk Vignettes [31] | 4 vignettes | Mental health professionals | Text (clinical vignettes) | Suicide risk |
| SuicidEmoji [32] | 25,051 | Reddit users | Text | Suicidal ideation |
| SWMH [33] | 54,412 | Reddit users | Text (posts) | Suicidal ideation, depression, anxiety, bipolar disorder |
| Thought Disorder Subset [34] | 51 | Patients with schizophrenia and healthy controls | Transcripts | Schizophrenia, thought disorders |
| TMDD [35] | 232,895 posts | Twitter users | Text, images | Depression |
| T-SID [33] | 4,800 | Twitter users | Text (posts) | Suicidal ideation, depression, PTSD |
| Twt-60Users [36] | 25,362 users | Twitter users | Text (posts) | Depression |
| UMD Suicidality Dataset [37] | 934 users | Reddit users | Text (posts) | Suicidal ideation |
| UMD Suicidality v2 [38] | 22,258 users | Reddit users | Text (posts) | Suicide risk |
| Student Smartphone Sensing Dataset [39] | 10 selected from 150 students | University students | Smartphone sensor data | General wellbeing, affective states |
| WU3D [40] | 32,570 users | Weibo users | Text, images, social behavior | Depression |

1. Wright SN, Anticevic A. Generative AI for precision neuroimaging biomarker development in psychiatry. Psychiatry Res. 2024 Sep;339:115955. PMID: 38909415. doi: 10.1016/j.psychres.2024.115955.
2. Hur JK, Heffner J, Feng GW, Joormann J, Rutledge RB. Language sentiment predicts changes in depressive symptoms. Proc Natl Acad Sci U S A. 2024 Sep 24;121(39):e2321321121. PMID: 39284070. doi: 10.1073/pnas.2321321121.
3. Lee C, Mohebbi M, O'Callaghan E, Winsberg M. Large Language Models Versus Expert Clinicians in Crisis Prediction Among Telemental Health Patients: Comparative Study. JMIR Ment Health. 2024 Aug 2;11:e58129. PMID: 38876484. doi: 10.2196/58129.
4. Garg M, Saxena C, Krishnan V, Joshi R, Saha S, Mago V, et al. Cams: An annotated corpus for causal analysis of mental health issues in social media posts. arXiv preprint arXiv:220704674. 2022.
5. Heinz MV, Bhattacharya S, Trudeau B, Quist R, Song SH, Lee CM, et al. Testing domain knowledge and risk of bias of a large-scale general artificial intelligence model in mental health. Digit Health. 2023 Jan-Dec;9:20552076231170499. PMID: 37101589. doi: 10.1177/20552076231170499.
6. Coppersmith G, Dredze M, Harman C, Hollingshead K, Mitchell M, editors. CLPsych 2015 shared task: Depression and PTSD on Twitter. Proceedings of the 2nd workshop on computational linguistics and clinical psychology: from linguistic signal to clinical reality; 2015.
7. Chim J, Tsakalidis A, Gkoumas D, Atzil-Slonim D, Ophir Y, Zirikly A, et al., editors. Overview of the clpsych 2024 shared task: Leveraging large language models to identify evidence of suicidality risk in online posts. Proceedings of the 9th Workshop on Computational Linguistics and Clinical Psychology (CLPsych 2024); 2024.
8. Gaur M, Alambo A, Sain JP, Kursuncu U, Thirunarayan K, Kavuluru R, et al., editors. Knowledge-aware assessment of severity of suicide risk for early intervention. The world wide web conference; 2019.
9. Gratch J, Artstein R, Lucas GM, Stratou G, Scherer S, Nazarian A, et al., editors. The distress analysis interview corpus of human and computer interviews. LREC; 2014: Reykjavik.
10. Pirina I, Çöltekin Ç, editors. Identifying depression on reddit: The effect of training data. Proceedings of the 2018 EMNLP workshop SMM4H: the 3rd social media mining for health applications workshop & shared task; 2018.
11. Naseem U, Dunn AG, Kim J, Khushi M, editors. Early identification of depression severity levels on reddit using ordinal classification. Proceedings of the ACM Web Conference 2022; 2022.

12. Turcan E, McKeown K. Dreaddit: A reddit dataset for stress analysis in social media. arXiv preprint arXiv:191100133. 2019.
13. Barnhill JW. DSM-5® clinical cases: American Psychiatric Pub; 2013. ISBN: 1585629979.
14. Shen Y, Yang H, Lin L, editors. Automatic depression detection: An emotional audio-textual corpus and a gru/bilstm-based model. ICASSP 2022-2022 IEEE International Conference on Acoustics, Speech and Signal Processing (ICASSP); 2022: IEEE.
15. Shin D, Kim H, Lee S, Cho Y, Jung W. Using Large Language Models to Detect Depression From User-Generated Diary Text Data as a Novel Approach in Digital Mental Health Screening: Instrument Validation Study. J Med Internet Res. 2024 Sep 18;26:e54617. PMID: 39292502. doi: 10.2196/54617.
16. DeVault D, Artstein R, Benn G, Dey T, Fast E, Gainer A, et al., editors. SimSensei Kiosk: A virtual human interviewer for healthcare decision support. Proceedings of the 2014 international conference on Autonomous agents and multi-agent systems; 2014.
17. Xu X, Zhang H, Sefidgar Y, Ren Y, Liu X, Seo W, et al. GLOBEM dataset: multi-year datasets for longitudinal human behavior modeling generalization. Advances in Neural Information Processing Systems. 2022;35:24655-92.
18. Garg M, Shahbandegan A, Chadha A, Mago V. An annotated dataset for explainable interpersonal risk factors of mental disturbance in social media posts. arXiv preprint arXiv:230518727. 2023.
19. Cimtay Y, Ekmekcioglu E, Caglar-Ozhan S. Cross-subject multimodal emotion recognition based on hybrid fusion. IEEE Access. 2020;8:168865-78.
20. Hayati MFM, Ali MAM, Rosli ANM, editors. Depression Detection on Malay Dialects Using GPT-3. 7th IEEE-EMBS Conference on Biomedical Engineering and Sciences, IECBES 2022 - Proceedings; 2022.
21. Zeng G, Yang W, Ju Z, Yang Y, Wang S, Zhang R, et al., editors. MedDialog: Large-scale medical dialogue datasets. Proceedings of the 2020 conference on empirical methods in natural language processing (EMNLP); 2020.
22. Cai H, Yuan Z, Gao Y, Sun S, Li N, Tian F, et al. A multi-modal open dataset for mental-disorder analysis. Scientific Data. 2022;9(1):178.
23. Zhou W, Prater LC, Goldstein EV, Mooney SJ. Identifying Rare Circumstances Preceding Female Firearm Suicides: Validating A Large Language Model Approach. JMIR Ment Health. 2023 Oct 17;10:e49359. PMID: 37847549. doi: 10.2196/49359.
24. Gouniai JM, Smith KD, Leonte KG. Do clergy recognize and respond appropriately to the many themes in obsessive-compulsive disorder?: Data from a Pacific Island community. Mental health, religion & culture. 2022;25(1):33-46.

25. Chen J, Ro T, Zhu Z. Emotion recognition with audio, video, EEG, and EMG: a dataset and baseline approaches. IEEE Access. 2022;10:13229-42.
26. Bauer B, Norel R, Leow A, Abi Rached Z, Wen B, Cecchi G. Using Large Language Models to Understand Suicidality in a Social Media–Based Taxonomy of Mental Health Disorders: Linguistic Analysis of Reddit Posts. JMIR Mental Health. 2024;11. doi: 10.2196/57234.
27. Radwan A, Amarneh M, Alawneh H, Ashqar HI, AlSobeh A, Magableh AAAR. Predictive analytics in mental health leveraging llm embeddings and machine learning models for social media analysis. International Journal of Web Services Research (IJWSR). 2024;21(1):1-22.
28. Sampath K, Durairaj T, editors. Data set creation and empirical analysis for detecting signs of depression from social media postings. International Conference on Computational Intelligence in Data Science; 2022: Springer.
29. Mauriello ML, Lincoln T, Hon G, Simon D, Jurafsky D, Paredes P, editors. Sad: A stress annotated dataset for recognizing everyday stressors in sms-like conversational systems. Extended abstracts of the 2021 CHI conference on human factors in computing systems; 2021.
30. Haque A, Reddi V, Giallanza T, editors. Deep learning for suicide and depression identification with unsupervised label correction. Artificial Neural Networks and Machine Learning–ICANN 2021: 30th International Conference on Artificial Neural Networks, Bratislava, Slovakia, September 14–17, 2021, Proceedings, Part V 30; 2021: Springer.
31. Levi-Belz Y, Gamliel E. The effect of perceived burdensomeness and thwarted belongingness on therapists' assessment of patients' suicide risk. Psychotherapy research. 2016;26(4):436-45.
32. Zhang T, Yang K, Ji S, Liu B, Xie Q, Ananiadou S. SuicidEmoji: Derived Emoji Dataset and Tasks for Suicide-Related Social Content. Proceedings of the 47th International ACM SIGIR Conference on Research and Development in Information Retrieval; Washington DC, USA: Association for Computing Machinery; 2024. p. 1136–41.
33. Ji S, Li X, Huang Z, Cambria E. Suicidal ideation and mental disorder detection with attentive relation networks. Neural Computing and Applications. 2022;34(13):10309-19.
34. Elvevåg B, Foltz PW, Weinberger DR, Goldberg TE. Quantifying incoherence in speech: an automated methodology and novel application to schizophrenia. Schizophrenia research. 2007;93(1-3):304-16.
35. Gui T, Zhu L, Zhang Q, Peng M, Zhou X, Ding K, et al., editors. Cooperative multimodal approach to depression detection in twitter. Proceedings of the AAAI conference on artificial intelligence; 2019.
36. Jamil Z. Monitoring tweets for depression to detect at-risk users: Université d'Ottawa/University of Ottawa; 2017.

37. Shing H-C, Nair S, Zirikly A, Friedenberg M, Daumé III H, Resnik P, editors. Expert, crowdsourced, and machine assessment of suicide risk via online postings. Proceedings of the fifth workshop on computational linguistics and clinical psychology: from keyboard to clinic; 2018.
38. Zirikly A, Resnik P, Uzuner O, Hollingshead K, editors. CLPsych 2019 shared task: Predicting the degree of suicide risk in Reddit posts. Proceedings of the sixth workshop on computational linguistics and clinical psychology; 2019.
39. Zhang T, Teng S, Jia H, D'Alfonso S. Leveraging LLMs to Predict Affective States via Smartphone Sensor Features. Companion of the 2024 on ACM International Joint Conference on Pervasive and Ubiquitous Computing; Melbourne VIC, Australia: Association for Computing Machinery; 2024. p. 709–16.
40. Wang Y, Wang Z, Li C, Zhang Y, Wang H. A multitask deep learning approach for user depression detection on sina weibo. arXiv preprint arXiv:200811708. 2020.
